# Physical–Chemical Coupling Coassembly Approach to Branched Magnetic Mesoporous Nanochains with Adjustable Surface Roughness

**DOI:** 10.1002/advs.202309564

**Published:** 2024-04-06

**Authors:** Xirui Huang, Minchao Liu, Qianqian Lu, Kexin Lv, Lipeng Wang, Sixing Yin, Minjia Yuan, Qi Li, Xiaomin Li, Tiancong Zhao, Dongyuan Zhao

**Affiliations:** ^1^ College of Chemistry and Materials Department of Chemistry Laboratory of Advanced Materials Shanghai Key Laboratory of Molecular Catalysis and Innovative Materials State Key Laboratory of Molecular Engineering of Polymers Collaborative Innovation Center of Chemistry for Energy Materials (2011‐iChEM) Fudan University Shanghai 200433 China; ^2^ Shanghai Qiran Biotechnology Co., Ltd Shanghai 201702 China

**Keywords:** bacterial inhibition, mesoporous material, multiple cooperative assembly processes, self‐assembly, tunable surface topology

## Abstract

Self‐assembly processes triggered by physical or chemical driving forces have been applied to fabricate hierarchical materials with subtle nanostructures. However, various physicochemical processes often interfere with each other, and their precise control has remained a great challenge. Here, in this paper, a rational synthesis of 1D magnetite‐chain and mesoporous‐silica‐nanorod (Fe_3_O_4_&mSiO_2_) branched magnetic nanochains via a physical–chemical coupling coassembly approach is reported. Magnetic‐field‐induced assembly of magnetite Fe_3_O_4_ nanoparticles and isotropic/anisotropic assembly of mesoporous silica are coupled to obtain the delicate 1D branched magnetic mesoporous nanochains. The nanochains with a length of 2–3 µm in length are composed of aligned Fe_3_O_4_@mSiO_2_ nanospheres with a diameter of 150 nm and sticked‐out 300 nm long mSiO_2_ branches. By properly coordinating the multiple assembly processes, the density and length of mSiO_2_ branches can well be adjusted. Because of the unique rough surface and length in correspondence to bacteria, the designed 1D Fe_3_O_4_&mSiO_2_ branched magnetic nanochains show strong bacterial adhesion and pressuring ability, performing bacterial inhibition over 60% at a low concentration (15 µg mL^−1^). This cooperative coassembly strategy deepens the understanding of the micro‐nanoscale assembly process and lays a foundation for the preparation of the assembly with adjustable surface structures and the subsequent construction of complex multilevel structures.

## Graphical abstract

A novel physical–chemical coupling coassembly approach is demonstrated for the synthesis of branched magnetic mesoporous nanochains with adjustable surface roughness.

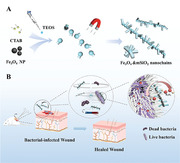

## 1 Introduction

As one of the most elegant bottom‐up methods, organizing nanoparticles into specific assembly structures can create superior properties.^[^ ^1^, ^2^, ^3^, ^4^, ^5^, ^6^, ^7^ ^]^ Over the past decades, a plethora of nano‐assemblies with various dimensions, morphologies, and compositions have been fabricated, which are based on methods including chemical linker‐assisted assembly, solvent evaporation‐driven assembly, spatial constraint assembly, and so on.^[^ ^8^, ^9^, ^10^, ^11^ ^]^ Among them, magnetic‐induced 1D nanoassemblies, which are formed by the alignment of magnetic particles under an applied magnetic field and subsequently fixed through sol‐gel chemistry to form interconnected layers, have attracted wide attention.^[^ ^12^, ^13^, ^14^ ^]^ Magnetic‐induced 1D nano‐assemblies possess advantages including easy fabrication, unique anisotropic properties, large aspect ratio, and good nano‐biological interface properties, and have shown excellent application prospects in the fields of sensors, radiation absorption, catalysts, energy materials, and biomedicines.^[^ ^15^, ^16^, ^17^, ^18^ ^]^ To date, however, the surface of the 1D assembly nanomaterials reported is usually smooth and low in roughness,^[^ ^13^, ^15^, ^19^ ^]^ but rarely are the design and regulation of hierarchical structures considered, especially the surface topological structures. This makes the nano‐assemblies difficult to adhere to and penetrate the biological host, hindering the performance greatly.^[^ ^20^, ^21^, ^22^ ^]^

To tune the surface topological structure of nanomaterials, several strategies have been conceived and developed.^[^ ^23^, ^24^, ^25^, ^26^, ^27^ ^]^ Among them, anisotropic assembly growth is the most controllable approach.^[^ ^20^, ^28^, ^29^, ^30^ ^]^ Various nanomaterials with surface roughness structures have been synthesized based on strategies such as epitaxial growth and surface dynamics‐induced multisite growth.^[^ ^31^, ^32^, ^33^, ^34^ ^]^ However, the application of this anisotropic growth method is currently limited to the construction of surface topological structures on individual nanoparticles, and no studies have so far reported introducing anisotropic growth during the nanoparticles’ self‐assembly process.

For the preparation of 1D nanochains using the method of external magnetic‐field‐directed assembly, additional precursors to coat onto the nanochain and “anchor” the assembly are crucial for the formation of controllable 1D nanochains. To generate 1D nanochain with rough surface structure, the precursor is required to perform two different roles: isotropic coating, and anisotropic growth, to anchor the nanochains and fabricate rough surfaces respectively. Meanwhile, these two modes are also mutually influenced by the assembly induced through a magnetic field, and the multiple factors in the synthesis process make it difficult to control the multiple physical–chemical coupling processes. Although there have been numerous reports about the synergistic assembly of multiple assembly processes, most of them focus on adjusting the reaction environment to coordinate various chemical assembly processes.^[^ ^35^, ^36^ ^]^ However, there is currently a lack of research on how to coordinate and achieve the physical–chemical coupled coassembly. Therefore, constructing 1D nanoassemblies with controllable surface topological structures is still a big challenge.

Here, in this work, we report a rational synthesis of 1D Fe_3_O_4_&mSiO_2_ branched magnetic nanochains via a physical–chemical coupling coassembly approach. By precisely coordinating magnetic assembly, mesoporous silica anisotropic growth, and isotropic silica coating process, we achieved 1D adjustable roughness magnetic mesoporous nanochains (Fe_3_O_4_&mSiO_2_ branched nanochains, denoted as BNCs). The main chain consists of 1D aligned 150 nm Fe_3_O_4_@mSiO_2_ nanospheres with mSiO_2_ branches on the chain (a length of 300 nm, a width of 220 nm). Chemical assembly and physical assembly mutually influence each other through the interfacial assembly process, ultimately achieving coordination and synergy, allowing them to proceed in a synchronized manner. By properly regulating several assembly processes, the density and length of the branches can be adjusted. The roughness can be adjusted from 0.15 to 8.5, achieving control of the surface topological structures. The length that matches the bacteria and the unique rough surface deliver large pressure to bacteria, resulting in high binding strength between BNCs and bacteria.^[^ ^22^, ^31^ ^]^ The magnetite Fe_3_O_4_ nanoparticles can catalyze the decomposition of H_2_O_2_ at a low concentration to generate ·OH through the Fenton reaction, resulting in oxidative damage to bacterial cell membranes.^[^ ^37^, ^38^, ^39^ ^]^ Strong adhesion and rotating magnetic field (RMF) enhanced diffusion of reactive oxygen species (ROS) render BNCs good antibacterial effects, performing a novel synergistic antibacterial platform. This study has developed a novel physical–chemical synergistic assembly process that balances the magnetic‐field‐induced assembly, isotropic and anisotropic nucleation, laying the foundation for the construction of new hierarchical nanomaterials.

## 2 Results and Discussion

**Figure 1:**
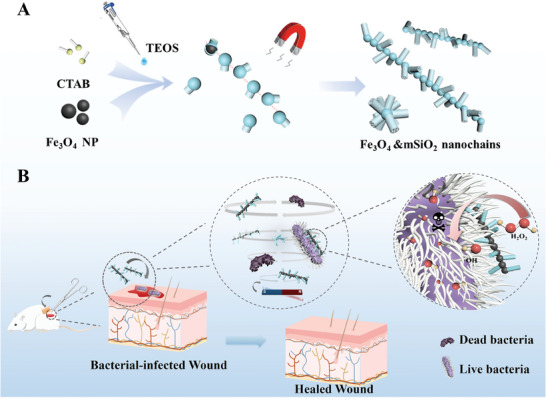
A) Schematic illustration of the physical–chemical coupling coassembly approach to 1D adjustable roughness magnetic mesoporous nanochains (Fe_3_O_4_&mSiO_2_ branched nanochains, denoted as BNCs). B) Schematic illustration of the antibacterial mechanism of BNCs for synergistic antibacterial therapy.

**Figure 2:**
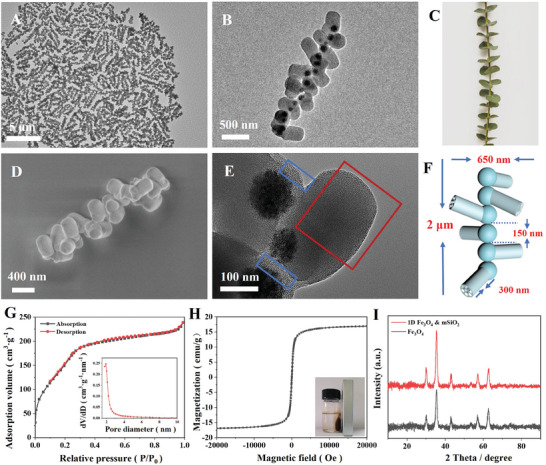
A,B) Transmission electron microscopy (TEM) images with different magnifications of 1D Fe_3_O_4_&mSiO_2_ branched nanochains (BNCs). C) Digital photo of a branch with leaves, demonstrating multi‐branch structure in nature. D) Scanning electron microscopy (SEM) image, E) high‐resolution transmission electron microscopy (HRTEM) image, F) 3D structural model, G) N_2_ adsorption–desorption isotherms, H) magnetic hysteresis loop, I) XRD pattern of BNCs. The pores in the red frame show highly ordered hexagonal mesostructure, while those in the blue frame marked area are disordered. The inset in (G) is the corresponding pore size distribution. The inset in (H) is the digital photo of BNCs adsorbed by a magnet.

Superparamagnetic Fe_3_O_4_ nanoparticles with a diameter of ≈120 nm are first prepared by the solvothermal method (Figure S1, Supporting Information).^[^ ^40^ ^]^ 1D branched magnetic mesoporous Fe_3_O_4_&mSiO_2_ nanochains (BNCs) can be fabricated via a physical–chemical coupling coassembly strategy in one step (**Figure** **1**A). Transmission electron microscopy (TEM) image shows the good dispersion and 1D branched structure of BNCs (**Figure** **2**A). TEM and scanning electron microscopy (SEM) images clearly demonstrate the 1D high roughness branched structure. The silica branches are random in orientation and separated in profile (Figure 2B–D), which resembles the branched structure in nature (Figure 2C and Figure S2, Supporting Information). The length of the obtained nanochains is measured to be 2.5 µm, while the diameter of the central chain is 150 nm (Figure 2F). Different elements (Fe, Si) in one nanochain can be identified by the energy dispersive spectrometer (EDS) mapping (Figure S3, Supporting Information), further demonstrating the 1D topology structure. High‐resolution transmission electron microscopy (HRTEM) images clearly reveal the pores on the mSiO_2_ branches and thick silica shells (≈30 nm) of the Fe_3_O_4_ nanoparticles (Figure 2E and Figure S4, Supporting Information). Nitrogen sorption isotherms (Figure 2G) of the obtained BNCs exhibit a representative type‐IV curve with a rapid increase of adsorption volume at a relative pressure of 0.2–0.4. The Brunauer−Emmett−Teller (BET) surface area and mesopore size are calculated to be 623 m^2^ g^−1^ and 2.88 nm (Figure 2G), respectively. Hysteresis loop experiment at 300 K shows that BNCs have superpara­magnetic properties with the magnetization saturation (Ms) value of 17.06 emu g^−1^ (Figure 2H). The digital photo (the inset of Figure 2H) suggests that the BNCs can be easily magnetized and manipulated by an applied magnetic field and lose their magnetization when the field is removed. The X‐ray diffraction (XRD) pattern also shows that the initial Fe_3_O_4_ particles are maintained very well after the anisotropic growth of mSiO_2_ branches and the assembly (Figure 2I).

Furthermore, we investigated the formation process of 1D branched magnetic mesoporous Fe_3_O_4_@mSiO_2_ nanochains. It can be observed that both the physical magnetic field and the chemical environment of the reaction significantly influence the outcome. The 1D branched mesoporous structure can be manipulated easily by controlling the time of the magnetic field applied during the reaction. When the magnetic field is introduced 4 min after the reaction begins, each Fe_3_O_4_ nanoparticle corresponds to at least one silica nanorod, with an average of 7 rods per 1 µm chain (Figures S5A and S6, Supporting Information). By advancing the introduction time of the magnetic field to 3 min, the number of the mesoporous silica rods is reduced to 5 rods per 1 µm chain (Figures S5B and S6, Supporting Information). Further advancing the introduction time of the magnetic field (2 min), the mSiO_2_ branches turn into wide bulges, resulting in a significant reduction in roughness (Figure S5C, Supporting Information). When the magnetic field is introduced much earlier, the nanoparticles appear to aggregate and form irregular structures (Figure S7, Supporting Information). On the other hand, when the magnetic field is applied too late after adding TEOS or no magnetic field is introduced, the Fe_3_O_4_ nanoparticles cannot assemble into chains, leaving only dispersed asymmetric nanoparticles (Figures S8 and S9, Supporting Information).

The ethanol content in the reaction solution also has a significant impact on the resulting products. When the reaction happens in pure water, only monodispersed Janus Fe_3_O_4_&mSiO_2_ nanoparticles can be formed even with the magnetic field applied (Figure S10, Supporting Information). At the same time, it can be observed that, unlike the BNCs whose Fe_3_O_4_ particles are coated with a layer of silica, the Fe_3_O_4_ particles are completely exposed in the obtained Janus Fe_3_O_4_&mSiO_2_ nanoparticles. When introducing ethanol into the reaction system (5 to 25 vol%), 1D branched mesoporous structures can be obtained (Figure S11, Supporting Information). The length and quantity of the mSiO_2_ nanorods decrease with increasing ethanol content, while the uniform silica coating on the chain surfaces gradually becomes thicker. Low roughness nanochain structures with few nanorods and thick silica shells can be obtained when increasing the ethanol content to 25%. By continuously increasing the ethanol amount (30%, 40%, 100%), only isolated core@shell structured Fe_3_O_4_@mSiO_2_ nanoparticles can be obtained, without anisotropic growth of mSiO_2_ (Figure S12, Supporting Information). Other factors including CTAB, TEOS, and application duration of magnetic fields can affect the length of mSiO_2_ nanorods and chains to some extent but do not significantly affect the formation of rough chains and the number of mSiO_2_ nanorods (Figures S13–S15, Supporting Information). Furthermore, the growth process of the mSiO_2_ is investigated in the reaction system by stopping the reaction before the magnetic field is introduced (Figure S16, Supporting Information). When the reaction is terminated 1 min after it starts, no nucleation process occurs and the surface of the Fe_3_O_4_ nanoparticles remains unchanged. Extending the reaction time to 2 min, a small amount of SiO_2_ oligomer deposition can be seen on the surface of the Fe_3_O_4_ nanoparticles. Further extending the reaction time (3 and 4 min), the coating layer gradually becomes thicker and the SiO_2_ nanorods gradually become longer.

Magnetic field, the anisotropic growth, and isotropic coating process of the silica oligomers coexist in the system, and the cooperation of the three assembly processes is crucial to the formation of BNCs and their topology structures. The magnetic field induces the 1D arrangement of Fe_3_O_4_ nanoparticles. The chemical environment determines the growth of silica, the isotropic coating process of silica acts as a “glue”, immobilizing the 1D arrangement of the Fe_3_O_4_ nanoparticles,^[^ ^8^, ^41^ ^]^ while the anisotropic growth of mesoporous silica nanorods forms the high‐roughness surface structure. The two processes are not independent. Rather, they can significantly influence each other. Based on the experimental data presented earlier, it is evident that both ethanol content and the time point of magnetic field exposure can influence the anisotropic assembly process of mesoporous silica, thereby altering the overall 1D branched mesoporous structure.

**Figure 3:**
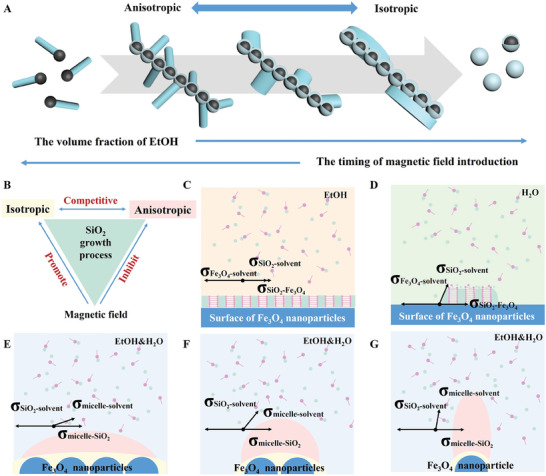
A) Schematic illustration of competition between isotropic and anisotropic growth of silane under different ethanol concentrations and different timing of magnetic field introduction during BNC synthesis. B) Schematic illustration of the correlation of three assembly processes in physical–chemical coupling coassembly. C,D) Schematic illustration of the nucleation of mSiO_2_ in different solvents. E–G) Schematic illustration of the influence of physical magnetic field on the interfacial anisotropic assembly process of mSiO_2_.

In this regard, we propose a physical–chemical coupling coassembly mechanism to explain the formation process of the 1D branched mesoporous structure. The chemical environment and the physical magnetic field influence the interfacial anisotropic assembly process of mesoporous silica through chemical potential and surface curvature, respectively, thus affecting the formation of the 1D branched mesoporous structure (**Figure** **3**A). The isotropic coating process and anisotropic growth have a competitive relationship, which is closely related to the ethanol concentration of the solution. The ethanol ratio affects the surface energy. According to the classical island growth mode of Volmer‐Weber and layer growth mode of Frank van der Merwe,^[^ ^42^, ^43^, ^44^ ^]^ the total surface energy change (*Δσ*) after the surface nucleation of CTAB/silicate micelles can be expressed as:

(1) $$\Delta \sigma \;=\left(\sigma_{Fe_{3}O_{4}-\mathrm{Si}O_{2}}+\sigma_{\mathrm{Si}O_{2}-\mathrm{solvent}}\right)\;-\sigma_{Fe_{3}O_{4}-\mathrm{solvent}}$$

where $\sigma_{Fe_{3}O_{4}-\mathrm{solvent}}\;$ and $\;\sigma_{\mathrm{Si}O_{2}-\mathrm{solvent}}\;$ are the surface energies of the Fe_3_O_4_ and SiO_2_ in the solvent, and $\sigma_{Fe_{3}O_{4}-\mathrm{Si}O_{2}}$ is the solid–solid interfacial energy between Fe_3_O_4_ and SiO_2_. The surface energy of the SiO_2_ ($\sigma_{\mathrm{Si}O_{2}-\mathrm{solvent}}$) increases as the volume percentage of H_2_O increases. When ethanol is used as solvent, the solid‐liquid interfacial energies of SiO_2_ are small, isotropic layer growth can dominate the growth of SiO_2_ on the Fe_3_O_4_ nanoparticles to form the core@shell structure (*Δσ* < 0, Figure 3C). In contrast, the solid–liquid interfacial energies of SiO_2_ increase greatly in water, which induces the anisotropic growth of the initial SiO_2_ nucleus (*Δσ* > 0, Figure 3D). Therefore, when the ethanol content is too low, no nanochain can be formed due to the lack of the isotropic nucleation process of silanes which are responsible for immobilizing the 1D arrangement of the Fe_3_O_4_ nanoparticles. On the other hand, excess ethanol can inhibit the anisotropic nucleation of silanes, thus hindering the construction of rough structures.

The magnetic field facilitates the assembly of the Fe_3_O_4_ nanoparticles, which affects its coordination with the silane nucleation. The onset of the magnetic field needs to be coordinated with the coating process of uniform nucleation so that the chain structure can be fixed by hydrolyzed cross‐linking of the silica precursors. If the magnetic field is introduced too early, the Fe_3_O_4_ particles have already formed long chains with a nearly smooth surface curvature, which inhibits the anisotropic nucleation of silanes due to the low curvature (Figure 3E). If the magnetic field introduction is delayed, the silane nucleate on the surface of multiple/single Fe_3_O_4_ nanoparticles, gradually increasing the surface curvature that needs to be overcome during nucleation, leading to the growth of silica rods with gradually increasing aspect ratios (Figure 3F). At the most appropriate timing, anisotropic growth of the mesoporous silica and 1D assembly of the Fe_3_O_4_ nanoparticles induced by the magnetic field occur simultaneously, thus do not affect each other. As a result, the obtained 1D branched mesoporous structure exhibits the highest number of silica rods. However, if the magnetic field is introduced too late, the nucleation of silanes has already been completed, and the isotropic coating of silica is inadequate to act as a “glue” to fix the obtained 1D nanochain. In such cases, only monodispersed asymmetric structures can be obtained. From this, we can conclude that in this system, isotropic nucleation and anisotropic nucleation of silane compete. The magnetic‐field‐induced assembly promotes isotropic nucleation of silane but inhibits anisotropic nucleation. Hence, the three assembly processes can be influenced simultaneously by physical and chemical factors. Only when these three processes reach a balance can a delicate 1D branched mesoporous structure be achieved through physical–chemical coupling coassembly.

**Figure 4:**
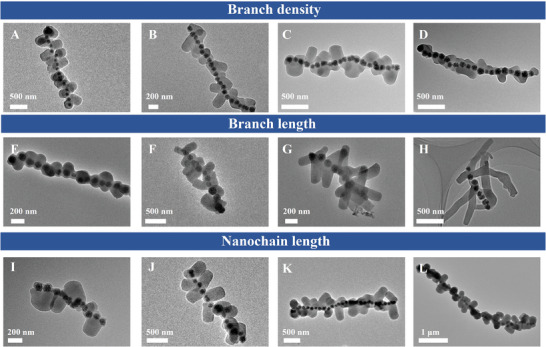
TEM images of various 1D Fe_3_O_4_&mSiO_2_ branched nanochains (BNCs) with different surface roughness. BNCs with different roughness share basically the same synthesis condition, with differences in: A–D) introduction time of magnetic field, where A) 4 min, B) 3.5 min, C) 3 min, D) 2 min. E–H) amount of TEOS and CTAB, where E) TEOS 180 µL, F) TEOS 300 µL, G) CTAB 150 mg, H) CTAB 200 mg; I–L) time duration of magnetic field, where I) 30 s, J) 60 s, K) 90 s, L) 120 s.

According to this physical–chemical coupling coassembly mechanism, we can manipulate the length and density of “branches” and the length of nanochains over a wide range, achieving precise control of the roughness and overall multilevel structures. Through regulation of magnetic field introduction time to modify the surface curvature of silane anisotropic assembly, the density of mSiO_2_ nanorods can be controlled from 7 rods per 1 µm chain to 3 rods per 1 µm (**Figure** **4**A–D). Simultaneously, as the introduction of the magnetic field is delayed, the length of the rods gradually increases, and the roughness of the BNCs is significantly enhanced (Figure S17, Supporting Information). If the physical–chemical coupling is kept unchanged so that the nucleation modality is consistent, we can regulate the length of the silica rods without changing their density. By regulating the amount of TEOS and CTAB, the length of mSiO_2_ nanorods can be controlled from 100 to 700 nm (Figure 4E–H). Moreover, reducing the water‐to‐alcohol ratio can also lead to an increase in the length of the rods and a decrease in shell thickness, thereby enhancing the roughness of the BNCs (Figure S18, Supporting Information). Furthermore, when both the introduction time of magnetic field and the chemical environment remain unchanged, mere regulation of the time duration of the applied magnetic field or the strength of magnetic field can affect the length of BNCs while not affecting the roughness (Figure S19, Supporting Information). When the magnetic field induction time is shortened (120 to 30 s), the nanochain length decreases from 4.5 to 1.2 µm (Figure 4I–L).

As one of the most important geometric parameters of nanoassemblies, the surface roughness (*R*a) of BNCs can be evaluated using the following Equation (2). The two most important parameters in the equation include: i) the ratio of mSiO_2_ branches to the Fe_3_O_4_ nanoparticles and ii) the length (*a*) and the width (*b*) of all branches, which can be well determined by TEM observation.

(2) $$Ra\;=\frac{n_{\mathrm{branch}}}{n_{\mathrm{nanoparticle}}}\;\times \;\frac{\frac{1}{n_{\mathrm{branch}}}\sum_{i\;=\;1}^{n}a}{\frac{1}{n_{\mathrm{branch}}}\sum_{i\;=\;1}^{n}b}=\frac{n_{\mathrm{branch}}\sum_{i=1}^{n}a}{n_{\mathrm{nanoparticle}}\sum_{i=1}^{n}b}$$

As shown in Figure 4, the architecture is highly controllable. According to the above calculation equation, the roughness can be adjusted from 0.15 (Figure 4D) to 8.5 (Figure 4H). To the best of our knowledge, this is the first time to achieve roughness regulation of such a large range and high controllability on 1D structures.

Bacterial infections pose a major threat to global health, afflicting millions of people annually.^[^ ^45^, ^46^ ^]^ Although burgeoning nanomaterials have emerged as a new generation of antibiotics for inhibiting bacteria, enhancing the interaction between nanomaterials and bacteria has been a difficult problem.^[^ ^47^ ^]^ The 1D branched structure of the BNCs matched with the natural bacilli, suggesting promising bacterial adhesion ability. Because the mesopore channels of mSiO_2_ coats facilitate mass transfer during the Fenton reaction process, the antibacterial efficacy of BNCs could also benefit due to the presence of Fe_3_O_4_ particles (Figure S20, Supporting Information).^[^ ^48^, ^49^, ^50^ ^]^ Therefore, we investigated the antibacterial activity of BNCs.

**Figure 5:**
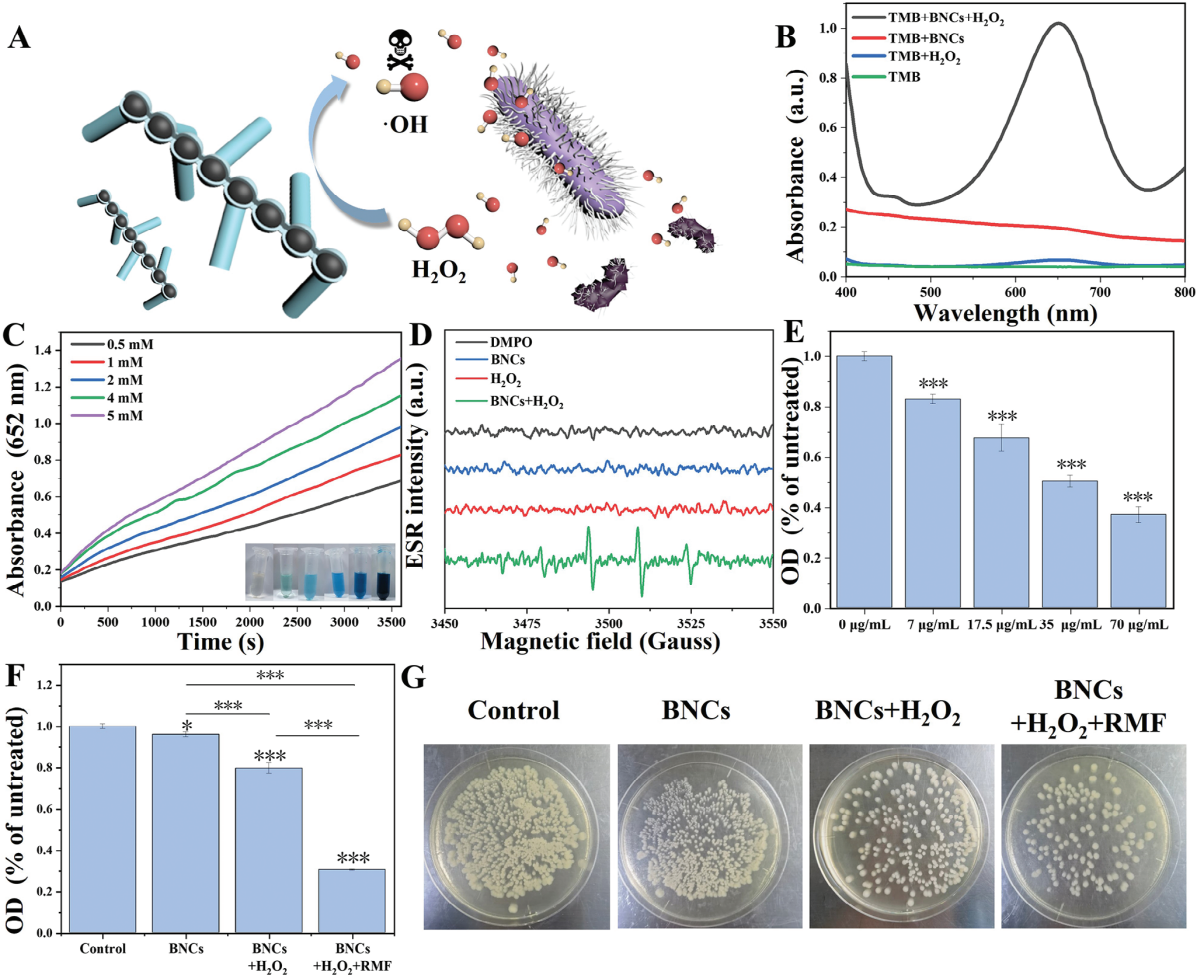
A) Schematic illustration of Fenton reaction based on 1D Fe_3_O_4_&mSiO_2_ branched nanochains (BNCs). B) UV–vis absorption spectra of the oxTMB products under catalysis of BNCs in the PBS buffer (pH 4.0). C) Time‐dependent absorbance changes at 652 nm of oxTMB products under the catalysis of BNCs with different H_2_O_2_ concentrations in the PBS buffer (pH 4.0). The inset in panel (C) is the photographs of the oxTMB products in the solution at different times under the catalysis of BNCs. D) ESR spectra in different conditions. E) OD_600_ values of the bacteria incubated with different concentrations of BNCs. Error bars are taken from three parallel tests per group. Data are expressed as mean standard ± s.d. (*n* = 3). F) OD600 values of bare bacteria and the bacteria incubated with BNCs, BNCs+ H_2_O_2_, and BNCs+ RMF+ H_2_O_2_ groups. Data are expressed as mean standard ± s.d. (*n* = 3). G) Evaluation of *E. coli* growth with different treatments by spread plate method. *p < 0.05, ***p < 0.001.

The catalytic performance of BNCs was first investigated. To examine the peroxidase‐like activity of the BNCs, the colorless 3,3′,5,5′‐tetramethylbenzidine (TMB), which displays a characteristic absorbance at 652 nm when converted to blue oxidized TMB (oxTMB), is used as the molecular probe to detect the production of ^•^OH in PBS solution (**Figure** **5**A).^[^ ^51^, ^52^, ^53^ ^]^ UV–vis absorbance spectra imply that BNCs can catalyze the oxidation of TMB via H_2_O_2_. In contrast, control experiments indicate that neither BNCs + TMB nor H_2_O_2_+ TMB generates the deep blue product oxTMB under our experimental conditions (Figure 5B). With the extension of reaction time, the color of the solution gradually deepens, indicating that the oxTMB concentration gradually increases. Furthermore, the catalytic activity is dependent on the concentration of H_2_O_2_ (Figure 5C and Figure S21, Supporting Information). Additionally, the ROS generation efficiency of BNCs with different roughnesses is essentially consistent, further indicating that the varying degrees of exposure of Fe_3_O_4_ particles do not affect the occurrence of the Fenton reaction (Figure S22, Supporting Information). This can be attributed to the unimpeded mass transfer facilitated by the covering mSiO_2_ shell layers. To further confirm the ROS species, the ^•^OH generation is detected by electron spin resonance (ESR) experiments. Free radical trapping agents also confirms the POD‐like activity of BNCs that converts H_2_O_2_ into ^•^OH radicals (Figure 5D). Subsequently, under the catalysis of BNCs with the presence of TMB and H_2_O_2_, the effect of RMF is investigated. The glycerol solution is used to simulate the wound with high viscosity. The uniform distribution of blue products oxTMB indicates that the rotating magnetic field accelerates the fast and uniform diffusion of the free radicals (Figure S23, Supporting Information). The rotation of BNCs under RMF can also be seen using an optical microscope (Figure S24, Supporting Information).

Encouraged by the matching size with bacteria, the rough shape structure, and the intrinsically enhanced peroxidase‐like activity, the antibacterial potential of BNCs is evaluated by growth‐inhibition assay in a liquid medium and the spread plate method. Gram‐negative *Escherichia coli* and Gram‐positive *Staphylococcus aureus* were used as models. First, we selected three representative morphologies of BNCs to determine their in vitro antibacterial efficiency, which are shown in Figure 4A,D,G, respectively. Obviously, BNCs can inhibit the growth of *E. coli* in LB medium in the presence of H_2_O_2_. BNCs with a roughness of 1.15 (Figures 2 and 4A) exhibit the highest antibacterial efficiency, as shown in Figure S25 (Supporting Information). In contrast, the antibacterial efficiency of smoother chains is limited. This difference likely arises because the smoother chains do not exert sufficient contact force with the bacteria (Figure S26, Supporting Information). The BNCs with a roughness of 1.15 are selected for further antibacterial and in vivo studies. With the increase in BNC concentration, the effect of bacterial inhibition is significantly enhanced (Figure 5E). The broad antibacterial activities against *E. coli* and *S. aureus* are investigated in four different treatment groups: I) PBS, II) BNCs, III) BNCs + H_2_O_2_, and IV) BNCs + H_2_O_2_ + RMF. As shown in Figure 5F, when the bacteria are treated with BNCs alone, the inhibition of bacterial growth is observed due to the topological interaction of the BNCs with the bacterial membrane. In addition, the BNCs + H_2_O_2_ group has limited inhibition efficiency. Expectedly, the introduction of RMF leads to the apparent reduction of bacterial survival percentages, respectively, implying that RMF effectively accelerates and enhances ROS diffusion. The results from the infrared thermal imaging at room temperature showed that there is no significant temperature change, thus ruling out the influence of magnetic heating effects (Figures S27 and S28, Supporting Information). The results from the plate counting method also confirm that the highest inhibition efficiency is achieved by the BNCs + H_2_O_2_ + RMF (Figure 5G). The antibacterial effect of BNCs on *S. aureus* is similar to that on E coli. When only BNCs and H_2_O_2_ are present, the inhibition efficiency can reach 59.5%. The introduction of RMF can significantly improve the inhibition efficiency, reaching 71% (Figure S29, Supporting Information). This further demonstrates the universality of the mechanical‐chemical synergy antibacterial mechanism against different types of bacteria. Moreover, the antibacterial effects of dispersed Fe_3_O_4_ nanoparticles and the pure SiO_2_ rough chains with etched Fe_3_O_4_ are far lower than those of BNCs (Figures S30 and S31, Supporting Information). The results revealed that the rough nanoassembled structure and the Fenton effect of Fe_3_O_4_ both play an indispensable role in the antibacterial process. To further decipher the excellent antibacterial capacity of the system, SEM was used to observe the bacterial morphological transformation. Untreated *E. coli* cells present a typical rod with a smooth surface and possess intact cell walls (Figure S32, Supporting Information). When exposed to the BNCs + H_2_O_2_ + RMF, the bacterial surface is severely damaged, owing to the generation and diffusion of H_2_O_2_ (Figure S33, Supporting Information). More importantly, SEM images also confirm that plenty of BNCs adhere to the surface of the bacteria, which characterizes physical interactions between the rough BNCs and the bacterial membranes.

**Figure 6:**
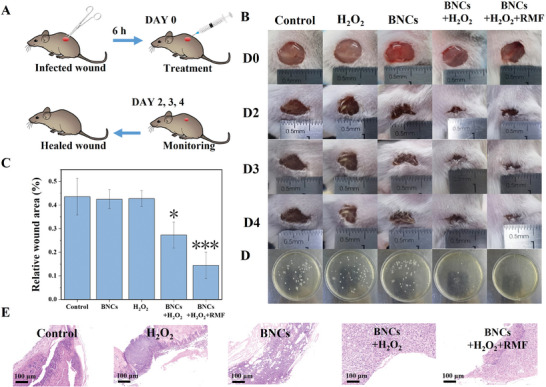
A) Schematic illustration of the wound healing process of the mice model. B) Photographs of *E. coli*‐infected wounds treated with different treatments. C) Percentage of wound area of different groups after 4 days. Data are expressed as mean standard ± s.d. (*n* = 3). D) Evaluation of *E. coli* separated from different wound tissue growth by spread plate method. E) Photographs showing sections of skin tissues with hematoxylin and eosin (H&E) staining after 4 d. *p < 0.05,***p < 0.001.

After confirming the excellent antibacterial property in vivo, we further assessed the antibacterial activity in skin wound models infected by *E. coli* in BALB/c mice (**Figure** **6**A). The mice are separated into five groups: treated with I) PBS, II) H_2_O_2_, III) BNCs, IV) BNCs + H_2_O_2_,and V) BNCs + H_2_O_2_ + RMF. Each group has three mice. Compared with controls, the wounds of mice treated with BNCs + H_2_O_2_ form scabs gradually. The BNCs + H_2_O_2_ + RMF group even forms scabs after three days of therapy and the scars become markedly smaller, implying that BNCs can prevent wound infection and the introduction of RMF can further accelerate the wound‐healing process (Figure 6B). The sizes of the wound areas of different treating groups are also calculated to monitor the healing condition. The area of the wounds treated with BNCs+ H_2_O_2_ + RMF is reduced to 14% of the original after four days, while the ratio of those treated only with BNCs or H_2_O_2_ is as high as 44% (Figure 6C). To further evaluate the sterilization efficiency, the number of bacteria on these wounds is quantified on the fourth day. As shown in Figure 6D, H_2_O_2_ at a low concentration has no antibacterial ability. The BNCs + H_2_O_2_ + RMF treatment leads to the most effective anti‐infection effect, which can thoroughly eradicate the live bacteria on the wound. The hematoxylin and eosin (H&E) staining is also used to evaluate wound healing ability. A great number of inflammatory cells and an incomplete epidermal layer are observed in the control group. On the other hand, in the BNCs+ H_2_O_2_ + RMF group, the epidermal structure of the wound is basically intact, with only a few inflammatory cells (Figure 6E). Furthermore, the main organs of the mice are examined by H&E staining, showing no apparent damage in these tissue slides (Figure S34, Supporting Information). Consequently, BNC materials present excellent antibacterial activity and further promote wound healing with good biocompatibility in vivo.

## 3 Conclusion

In summary, we demonstrate a brand‐new physical–chemical coupling coassembly strategy to direct the growth of 1D Fe_3_O_4_&mSiO_2_ nanochains with adjustable roughness surface structures. We illustrate how magnetic and chemical environments affect magnetic nanoparticle assembly and silane nucleation, and how physical–chemical assembly processes interact and coordinate. Nanochains of 2–3 µm with branches of 300 nm can be synthesized controllably, and so can monodisperse asymmetric particles, smooth chains and rough chains. The roughness can be modulated within 0.15–8.5. Due to the matching size with bacteria and the high surface roughness structure enhanced nano‐biointeraction, the Fe_3_O_4_&mSiO_2_ nanochains demonstrate excellent antibacterial properties and perform bacterial inhibition over 60% at a low concentration (15 µg mL^−1^). The *E. coli*‐infected wound model reveals that the BNCs+ H_2_O_2_ + RMF treatment leads to the most effective anti‐infection effect, and the area of the wound is reduced to 14% of the original after 4 d. We consider that the physical–chemical coupling coassembly strategy reveals how physical assembly and chemical assembly mutually influence and coordinate with each other, providing a pathway in the construction of new hierarchical nanomaterials with controllable surface topological structures.

## 4 Experimental Section

### Chemicals and Materials

Iron (III) chloride hexahydrate (FeCl_3_·H_2_O, AR), trisodium citrate (AR), sodium acetate trihydrate (AR), hexadecyltrimethylammonium bromide (CTAB, 99%), tetraethyl orthosilicate (TEOS, AR), 3,3′,5,5′‐tetramethylbenzidine (TMB), ammonium hydroxide solution (28 wt% NH_3_ in H_2_O), and ethanol were purchased from Sinopharm Chemical Reagent Co., Ltd. All chemicals in this work were used as received without further purification. Deionized water (18.2 MΩ cm, 25 °C) was used in all experiments, and all solutions were freshly prepared for immediate use in each experiment.

### Synthesis of Fe_3_O_4_ Nanoparticles

The Fe_3_O_4_ nanoparticles were synthesized according to the hydrothermal method reported previously.^[^ ^40^ ^]^ 3.250 g of FeCl_3_·H_2_O, 100.0 mL of ethylene glycol, 1.300 g of trisodium citrate, and 6.000 g of sodium acetate trihydrate were mixed and sonicated for 1 h. The mixture was then transferred into a 200.0 mL Teflon‐lined stainless‐steel autoclave. The autoclave was heated at 200 °C and maintained for 10 h. After cooling down to room temperature, the products were washed with distilled water and ethanol.

### Synthesis of 1D Fe_3_O_4_&mSiO_2_ Branched Magnetic Nanochains

In a typical synthesis, 30.0 mg of Fe_3_O_4_ nanoparticles were redispersed in a mixed solution containing CTAB (100.0 mg, 0.31 mmol), deionized water (20.0 mL), and ethanol (5.0 mL). The mixture was sonicated for 30 min. Subsequently, concentrated ammonia solution (1.0 mL, 28 wt%) was added, and the mixed dispersion was mechanically stirred (250 rpm) for 5 min. After that, 0.33 mL (0.30 g) of TEOS was injected into the solution. After the reaction for 4 min, the solution was exposed to the magnetic field for 90 s to induce the alignment of Fe_3_O_4_ particles. After the reaction for 3 h, the product was separated with a magnet and washed with ethanol and water three times, respectively. Finally, the obtained nanochains were redispersed in 60 mL of ethanol at 80 °C to remove the CTAB templates. The extraction was repeated twice, followed by washing thoroughly mesoporous nanochains were washed with ethanol.

### Analysis of Hydroxyl Radical (^•^OH) Generation

To evaluate the Fenton activities of the BNCs, 3,3′,5,5′‐tetramethylbenzidine (TMB) was employed as an indicator to monitor the ^•^OH production in PBS solutions (pH 4.0 or pH 5.5). The generation rates of oxTMB (determined by the absorption at 652 nm) can be converted into velocities of ^•^OH generation via the Lambert‐Beer law. In detail, 1 mL aqueous solution containing TMB (1 × 10^−3^ m), BNCs (100 µg mL^−1^), and a series of H_2_O_2_ concentrations (0.5 × 10^−3^, 1.0 × 10^−3^, 2.0 × 10^−3^, 4.0 × 10^−3^, and 5.0 × 10^−3^ m) was incubated at room temperature.

The generation of ^•^OH was also evaluated by an ESR spectroscopy spectrometer using DMPO spin‐trapping adduct. In the experiment, DMPO's concentration was 50 × 10^−3^ m. All mixtures were dispersed in PBS buffer (pH 4.0). The solutions were aspirated into quartz capillaries for ESR analysis.

### In Vitro Antibacterial Experiment

The Spread plate method was employed to assess the antibacterial ability of BNCs. Gram‐negative bacteria (*E. coli*), Gram‐positive (*S. aureus*) were treated with BNCs in four different groups: BNCs, BNCs+ H_2_O_2_, and BNCs+ RMF+ H_2_O_2_ groups, respectively. The bacterial solution was incubated with different concentrations of BNCs with H_2_O_2_ (5 × 10^−3^ m) that were dispersed in LB culture at 37 °C under orbital shaking at a speed of 180 rpm for 12 h. In the BNCs+RMF+H_2_O_2_ group, after coincubating with RMF for 30 min, the magnetic field was removed. The absorbance at 600 nm was recorded. OD (% of untreated) was acquired using the following equation:

(3) $$\mathrm{OD}\;\;\left(\%\;\mathrm{of}\;\mathrm{untreated}\right)=\frac{OD_{1}-OD_{\mathrm{Fe}3O4}}{OD_{0}}\;\times 100$$

In which, OD_1_ represents the OD_600_ value of the experimental group, OD_0_ represents the OD_600_ value of the control group.

### Morphological Characterization of Bacteria

For SEM images of bacteria, after the material absorbing bacteria for 6 h, the bacteria cultured at 37 °C were harvested by centrifugation at 3500 rpm for 5 min. They were washed with PBS and glutaraldehyde (2.5%) for 15 min. The bacteria were further washed with a series of ethanol (30%, 50%, 70%, 85%, 95%, and 100%). The bacteria in 100% ethanol were finally dried in a vacuum drying chamber at room temperature. Before imaging, the bacteria were sputter‐coated with platinum.^[^ ^54^ ^]^

### In Vivo Antibacterial Experiment

Animal procedures were in agreement with the guidelines of the Institutional Animal Care and Use Committee of Fudan University and performed in accordance with the institutional guidelines for animal handling. To evaluate the antibacterial potential of our designed system for treating wound infection, the injury model was built on the back of mice.^[^ ^51^ ^]^ Balby mice's back (6−8 weeks) was slashed and injected with 1 × 10^6^ *E. coli* cells to build the infected wound model. The mice were divided into four groups (three mice in each group). The mice were treated with I) PBS, II) H_2_O_2_, III) BNCs, IV) BNCs + H_2_O_2_,and V) BNCs + H_2_O_2_ + RMF on their wound in different groups. The wounds were observed and photographed. After 4 d of treatment, the mice were sacrificed, and the wound tissues were harvested. The wound tissues were placed in 1 mL of sterile saline and homogenized. The animal studies were conducted in compliance with the Institutional Animal Care and Use Committee guidelines.

### Characterization

Transmission electron microscopy (TEM), high‐resolution transmission electron microscopy (HRTEM), and high‐angle annular dark field imaging in the scanning TEM (HAADF‐STEM) observations were acquired on JEM‐2100F transmission electron microscope with an accelerating voltage of 200 kV equipped with a post‐column Gatan imaging filter (GIF‐Tridium). The samples for TEM measurements were suspended in ethanol and supported onto a carbon film on a Cu grid. Energy‐dispersive X‐ray spectroscopy (EDX) and energy‐dispersive X‐ray spectroscopy element mapping were performed on a JEM 2100F EDX instrument. X‐ray diffraction (XRD) patterns were collected by a Bruker D8 powder X‐ray diffractometer (Germany) with Ni‐filtered Cu Kα radiation (40 kV, 40 mA). Scanning electron microscopy (SEM) images were taken using a Hitachi S‐4800 ultrahigh resolution cold FEG with an in‐lens electron optic operating at 20 kV. Nitrogen adsorption‐desorption measurements were conducted to obtain information on the porosity. The measurements were conducted at 77 K with ASAP 2420 and Micromeritics Tristar 3020 analyzer (USA).

### Statistical Analysis

The experiment data were presented as means ± standard deviation, where they were repeated at least three times. For animal experiments, mice were randomly selected and analyzed as biological replicates. The normality of the data was tested using the Shapiro‐Wilk normality test. Data with normal distribution were analyzed by one‐way ANOVA to determine the significance of the difference as described in the figure legends. In all tests, the statistical significance for the tests was set at *p < 0.05, **p < 0.01 and ***p < 0.001. Data were analyzed using OriginPro 2018 C software.

## Conflict of Interest

The authors declare no conflict of interest.

## Supporting information

Supporting Information

## Data Availability

The data that support the findings of this study are available in the Supporting Information of this article.
