# Supplementary material for: Physical–Chemical Coupling Coassembly Approach to Branched Magnetic Mesoporous Nanochains with Adjustable Surface Roughness
Source: Adv Sci (Weinh). 2024 Apr 6;11(23):2309564. doi: 10.1002/advs.202309564 (PMC11187885; doi:10.1002/advs.202309564)
Supplement: Supplementary file 1 — Supporting Information [file ADVS-11-2309564-s001.docx]

Supporting Information

**Physical-chemical Coupling Co-Assembly Approach to Branched Magnetic Mesoporous Nanochains with Adjustable Surface Roughness**

*Xirui Huang^a^, Minchao Liu^a^, Qianqian Lu^a^, Kexin Lv^a^, Lipeng Wang^a^, Sixing Yin^a^, Minjia Yuan^b^, Qi Li^b^, Xiaomin Li^a^, Tiancong Zhao^a^*, and Dongyuan Zhao^a^*

**
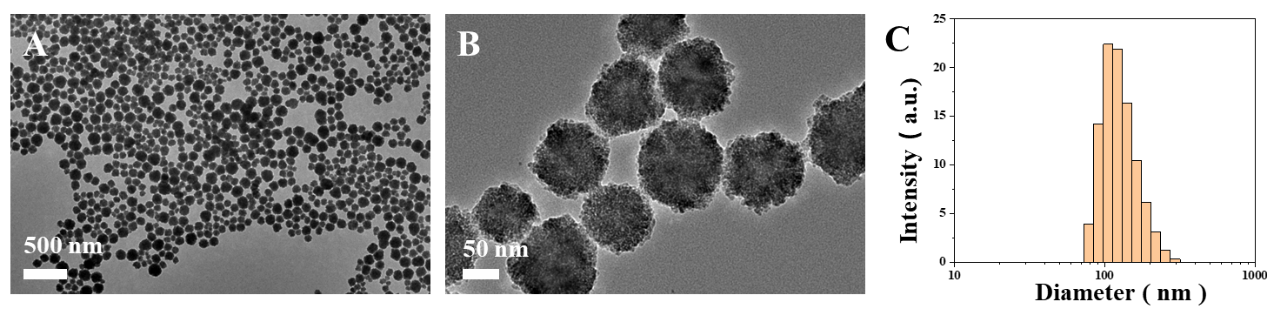
Figure S1.** (A, B) TEM images with different magnifications and (C) hydrodynamic diameters (determined by DLS) of Fe_3_O_4_ particles synthesized via a solvothermal reaction.^[1]^ The nanoparticles are uniformly distributed and have an average diameter of ≈ 120 nm.

**
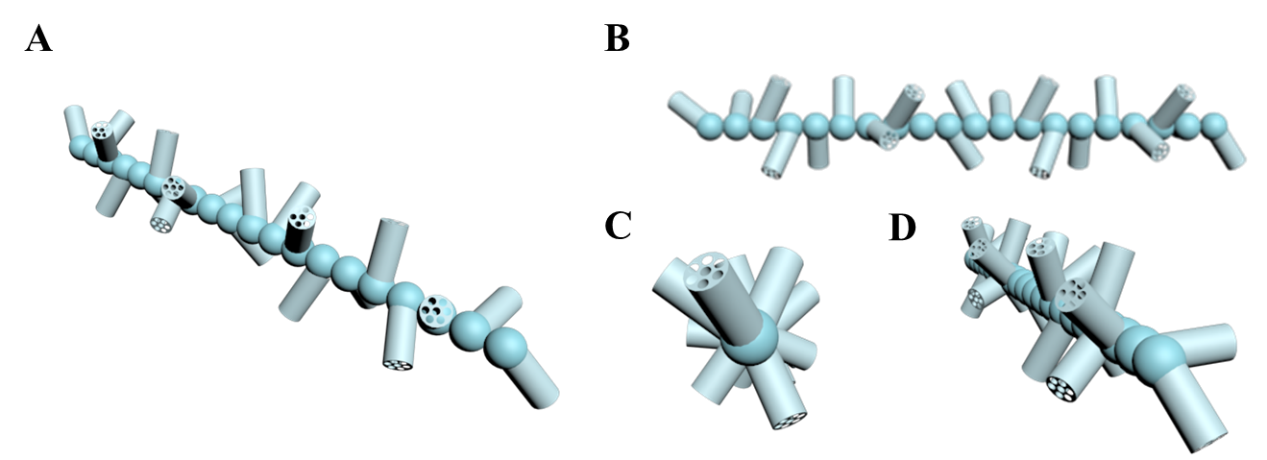
Figure S2.** 3D structural model and three views of Fe_3_O_4_&mSiO_2_ branched nanochains, indicating the 1D high roughness branched structure.

**
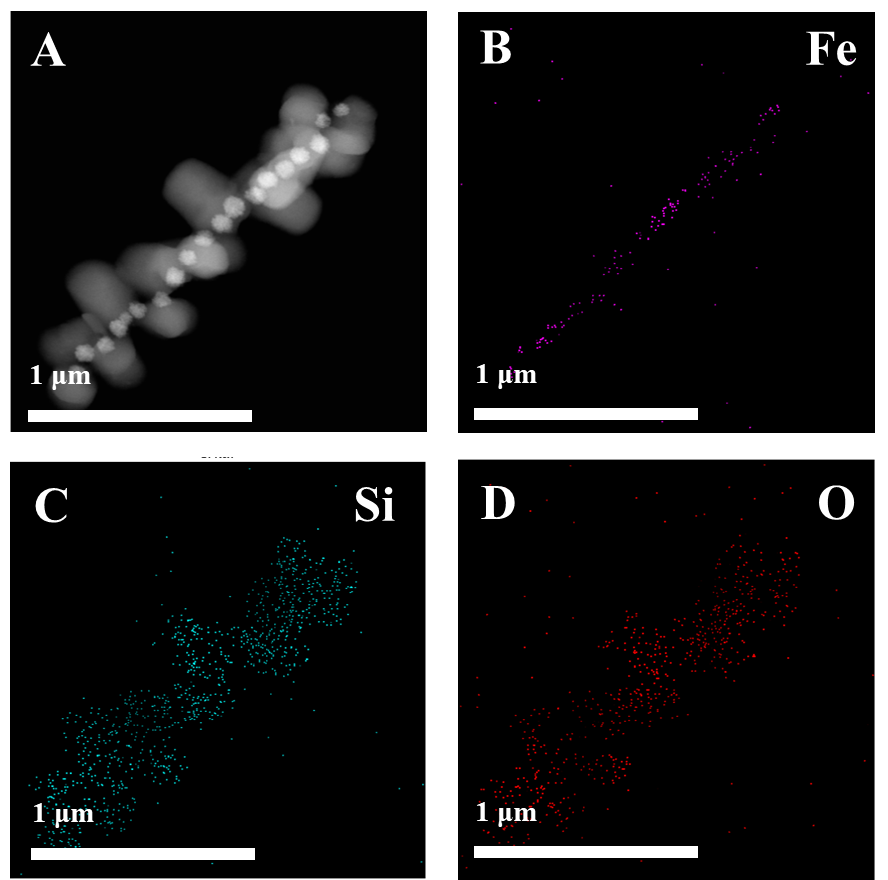
Figure S3.** (A) Dark field TEM image and (B-D) element mappings of Fe_3_O_4_&mSiO_2_ branched nanochains, indicating the 1D topology structure. The Fe_3_O_4_ nanoparticles are arranged in one dimension.

**
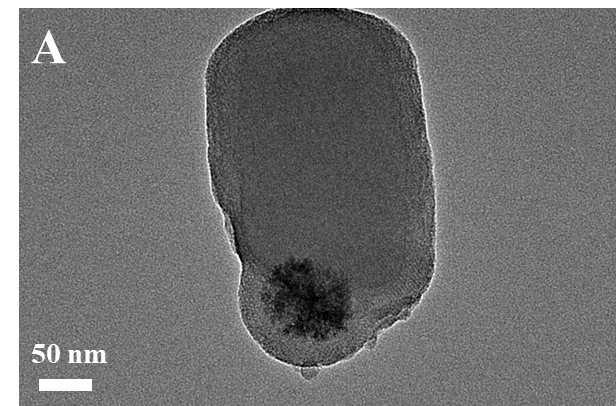
Figure S4.** TEM image of the individual Fe_3_O_4_&mSiO_2_ nanoparticle that is not involved in the assembly. The length of the mSiO_2_ branch is about 300 nm and there is a 30 nm thick silica shell on the Fe_3_O_4_ nanoparticle. The structure is consistent with the individual branch in Fe_3_O_4_&mSiO_2_ nanochains.

**
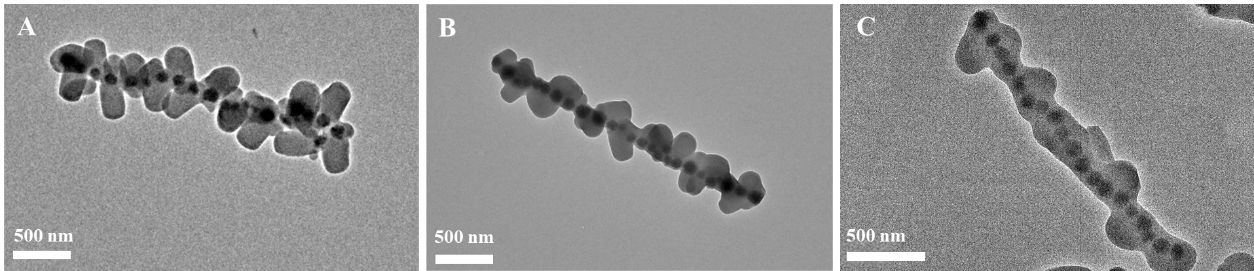
Figure S5.** TEM images of the as-prepared Fe_3_O_4_&mSiO_2_ samples with magnetic fields applied after different times of reaction: (A) 4 min; (B) 3 min; (C) 2 min. The 1D branched mesoporous structure can be easily manipulated by controlling the time of the magnetic field applied during the reaction. The density of mSiO_2_ nanorods increases with the delay of magnetic field introduction.

**
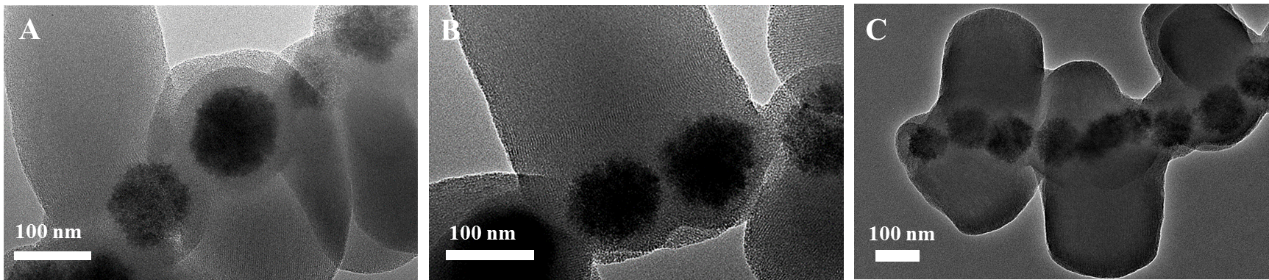
Figure S6.** High-resolution transmission electron microscopy (HRTEM) images of the as-prepared Fe_3_O_4_&mSiO_2_ samples with magnetic fields applied after different times of reaction: (A) 4 min; (B) 3.5 min; (C) 3 min. When the magnetic field introduction is advanced, silane nucleate simultaneously on the surface of multiple Fe_3_O_4_ nanoparticles, leading to the growth of silica rods with gradually decreasing aspect ratios.

**
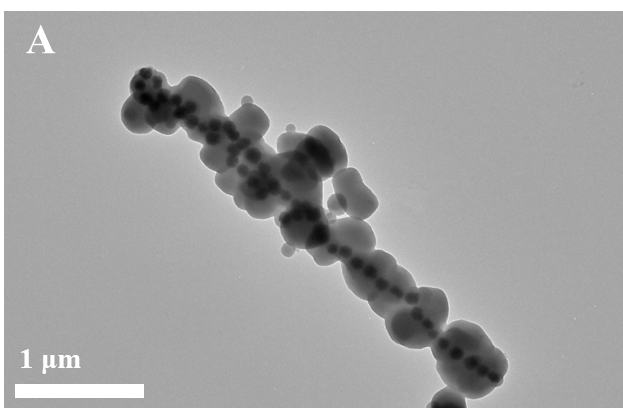
Figure S7.** TEM image of the as-prepared Fe_3_O_4_&mSiO_2_ samples with the magnetic field applied after 1.5 min of reaction. The Fe_3_O_4_ nanoparticles appear to aggregate and form a multi-chain structure.

**
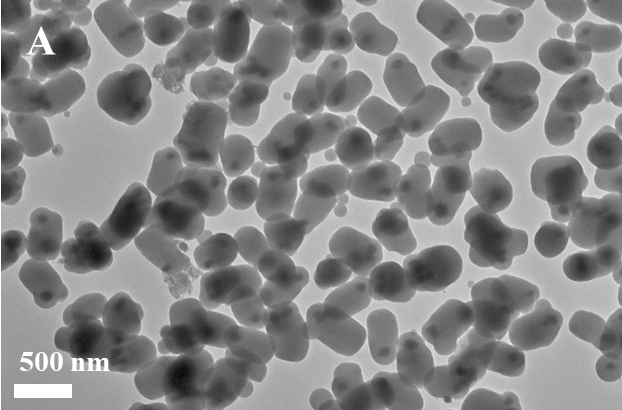
Figure S8.** TEM image of the as-prepared Fe_3_O_4_&mSiO_2_ samples with the magnetic field applied after 6 min of reaction. The Fe_3_O_4_ nanoparticles cannot assemble into chains, leaving only dispersed asymmetric nanoparticles. This result further evidences the beginning time of magnetically induced self-assembly needs to be earlier than the end of the coating process so that the chain structure can be fixed by hydrolyzed cross-linking of the silica precursor.

**
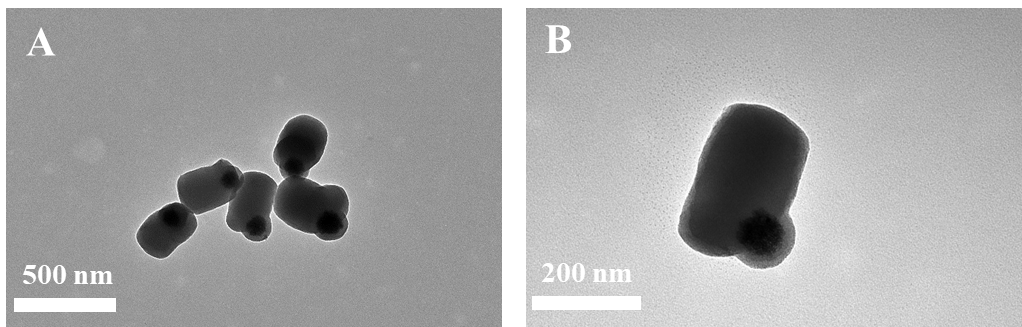
Figure S9.** TEM images of the as-prepared Fe_3_O_4_&mSiO_2_ samples without applied magnetic fields. Without magnetic field induction, the Fe_3_O_4_ nanoparticles cannot form 1D arrangements.

**Figure S10.** TEM images of the as-prepared Fe_3_O_4_&mSiO_2_ samples field in pure water and fabricated (A, B) without a magnetic field or that the magnetic fields are applied (C) after 1 min reaction; (D) after 2 min reaction. It is suggested that without the presence of ethanol in the system, the Fe_3_O_4_ nanoparticles cannot be linked into chains.
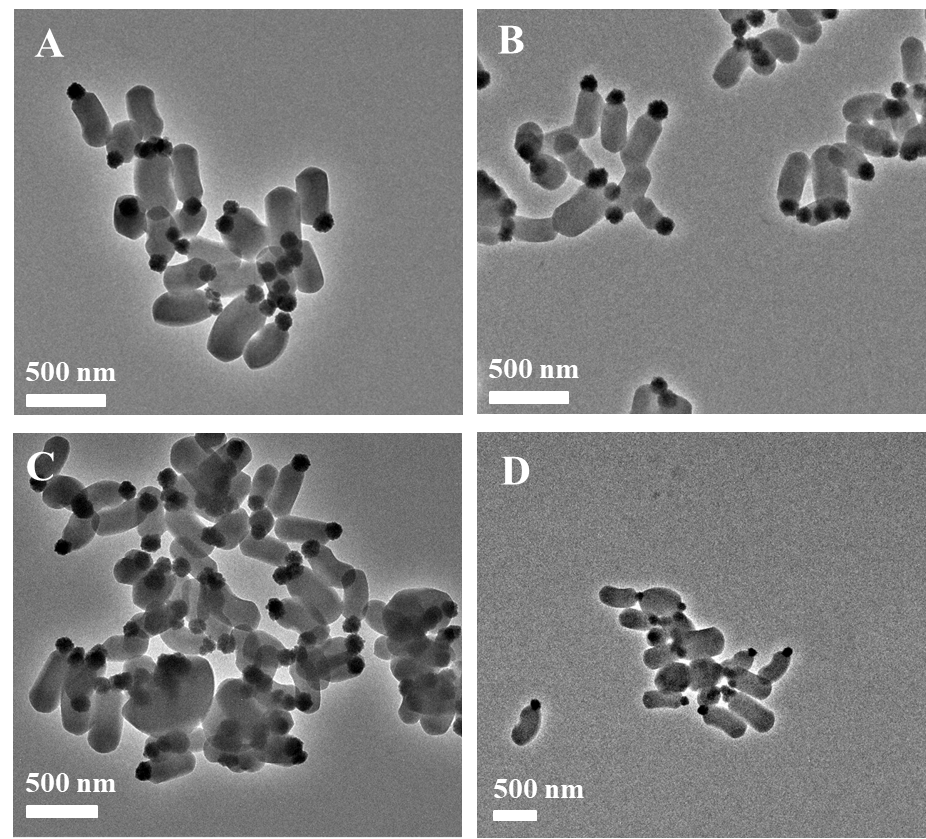


**
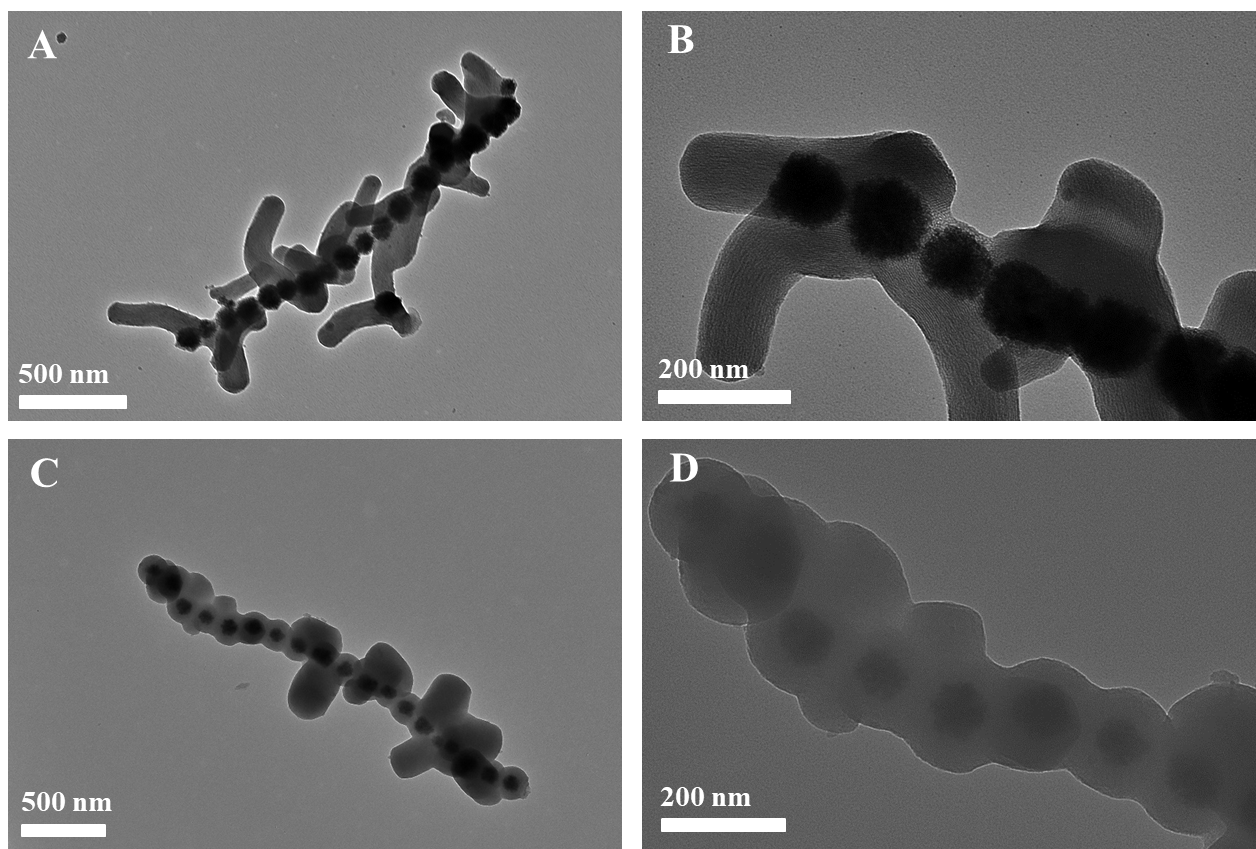
Figure S11.** TEM images of the as-prepared Fe_3_O_4_&mSiO_2_ samples using different volume fractions of ethanol (v/v%): (A) 5 %; (B) 25%. When a little ethanol is present in the reaction system (5 volume %), the homogeneous silica layers are thin, and very long nanorods can be obtained. As a comparison, with the magnetic field introduced at the same time, a low roughness nanochain structure with fewer nanorods and thicker silica shells can be obtained when the ethanol content is increased to 25%.

**
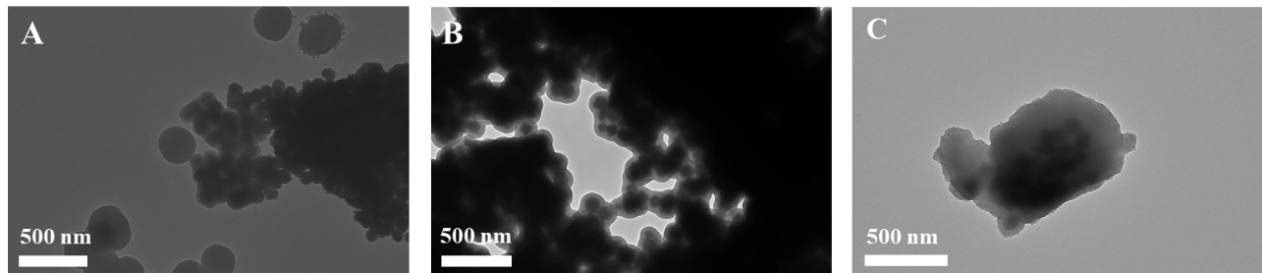
Figure S12.** TEM images of the as-prepared Fe_3_O_4_&mSiO_2_ samples using different volume fractions of ethanol (v/v%): (A) 30 %; (B) 40 %; (C) 100 %. Only uniformly coated Fe_3_O_4_ nanoparticles can be obtained. The irregular aggregation also occurs because of the induced magnetic field.

**
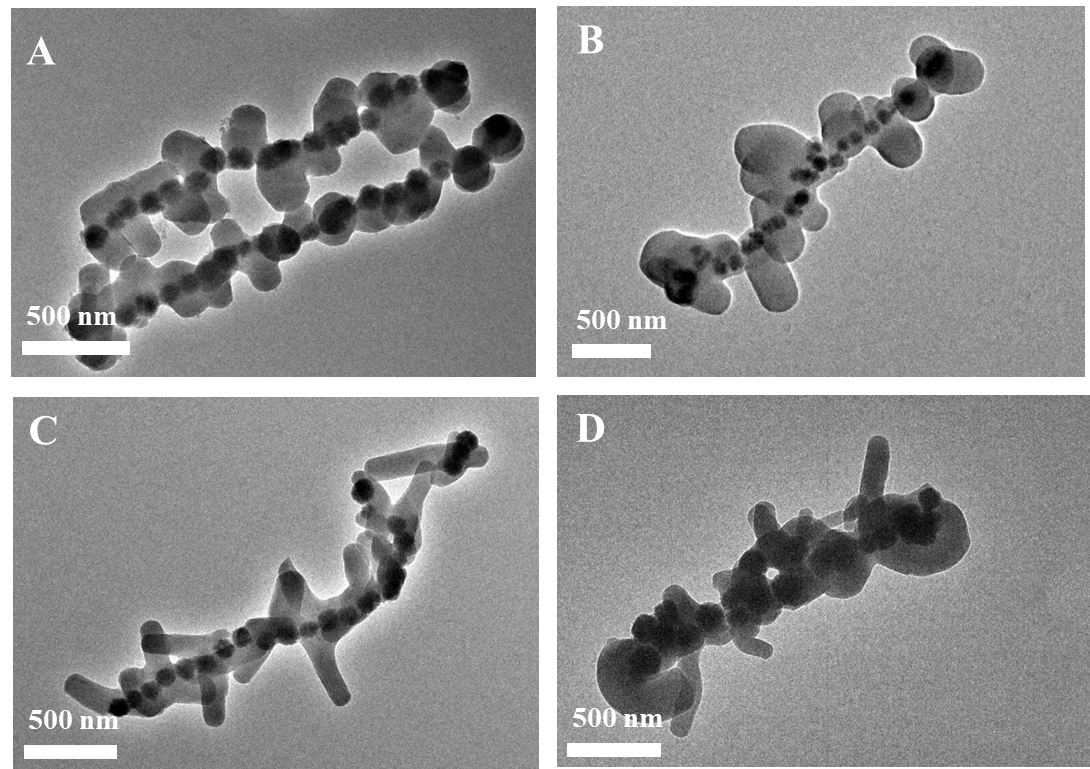
Figure S13.** TEM images of the as-prepared Fe_3_O_4_&mSiO_2_ samples using different amounts of CTAB: (A) 50 mg; (B) 100 mg; (C) 150 mg; (D) 200 mg. Results demonstrate that the amount of CTAB plays an important role in the length and morphology of branches. With the increase of CTAB, the width of nanorods gradually decreases and the length increases. When the CTAB amount reaches 200 mg, mSiO_2_ nanorods begin to wind the nanoparticles. This result can be explained by the rod-shaped micelles presenting a “lie down” manner under a high CTAB concentration, parallel to the surface of the Fe_3_O_4_ nanoparticle.

**
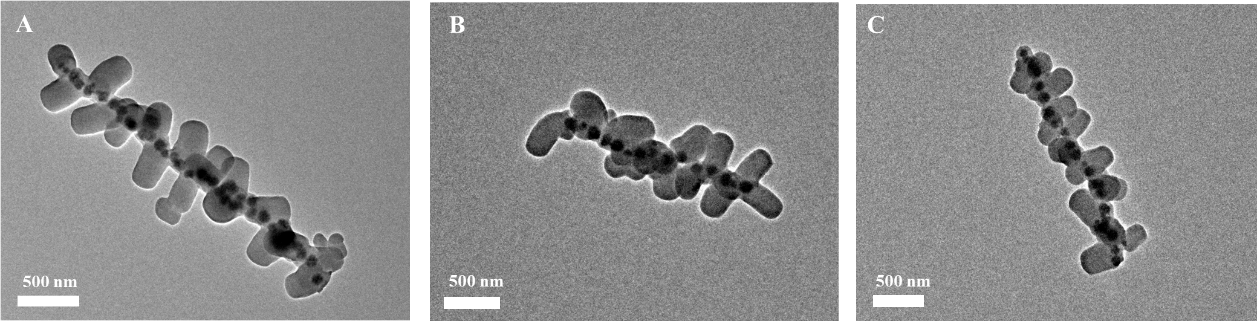
Figure S14.** TEM images of the as-prepared Fe_3_O_4_&mSiO_2_ samples using different amounts of TEOS. (A) 240 μL; (B) 300 μL; (C) 360 μL. The amount of TEOS can influence the length of the mSiO_2_ branches.

**
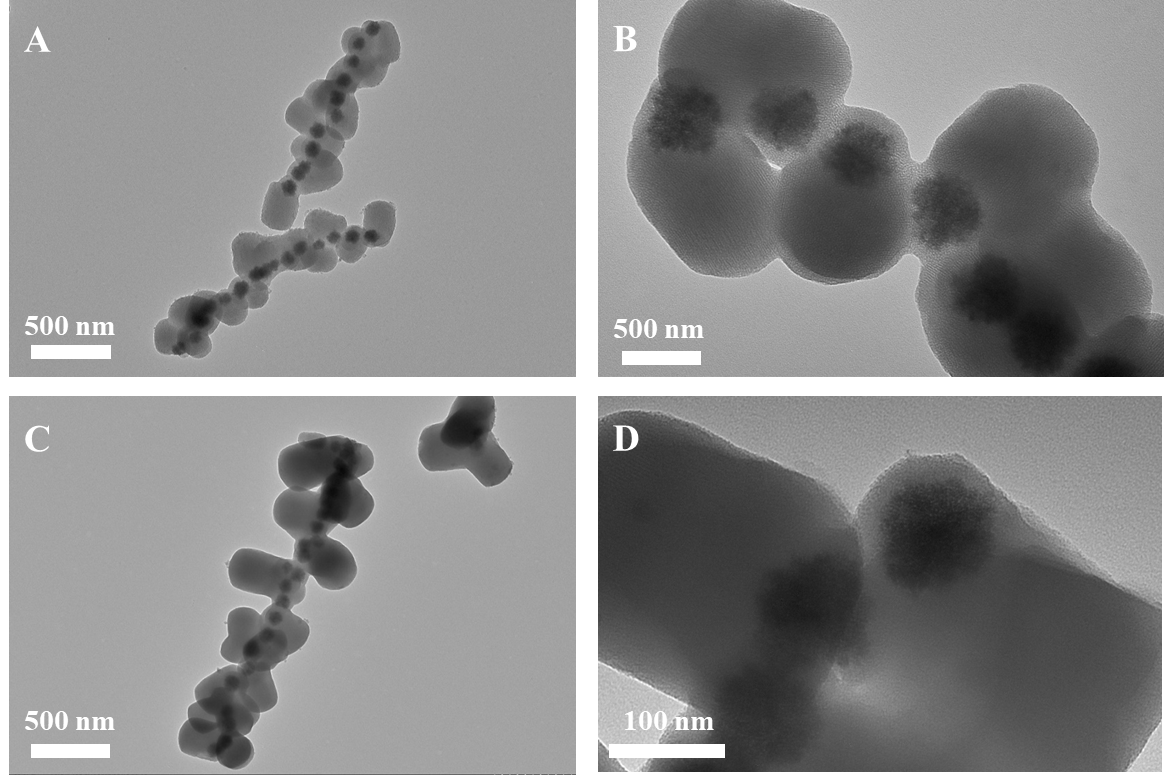
Figure S15.** TEM images of the as-prepared Fe_3_O_4_&mSiO_2_ samples with the magnetic field applied for a duration of (A, B) 30 s; (C, D) 1 min. The time duration of the applied magnetic field can affect the length of Fe_3_O_4_&mSiO_2_ nanochains.

**
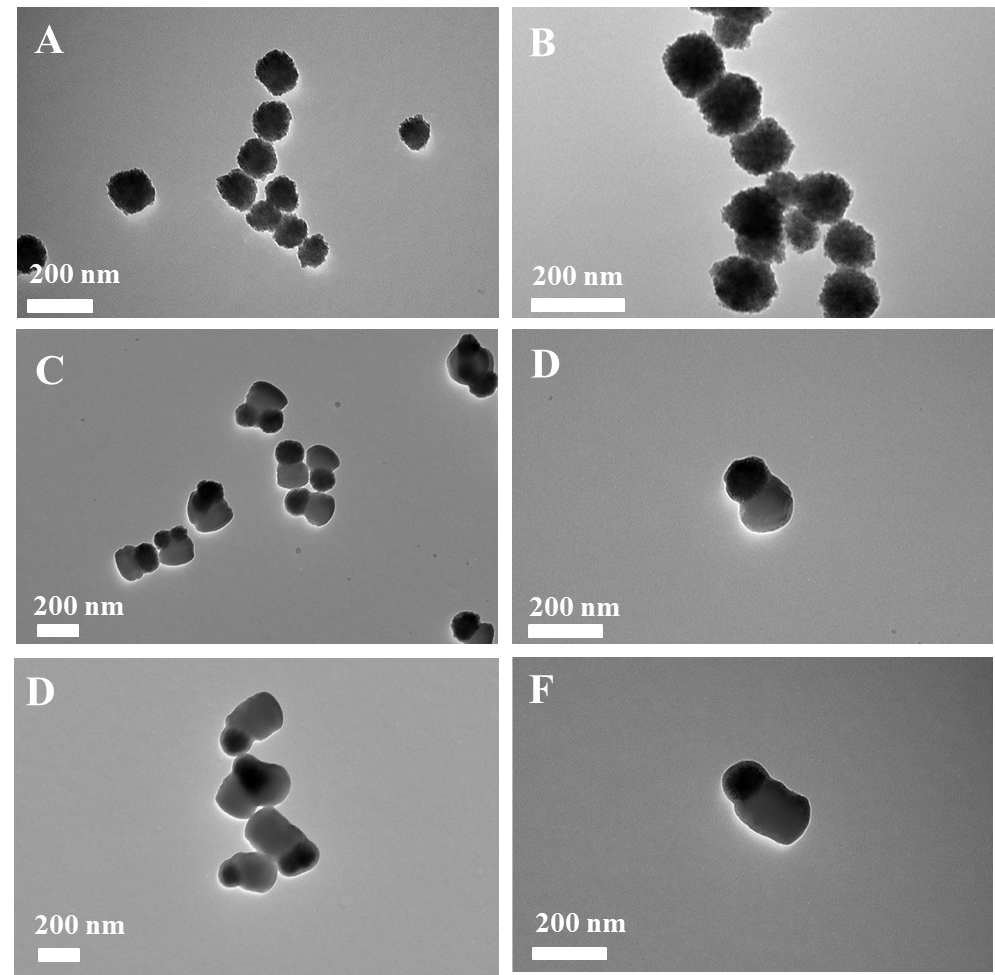
Figure S16.** TEM images of the as-prepared Fe_3_O_4_&mSiO_2_ samples. The reaction is terminated after (A) 1 min; (B) 2 min; (C, D) 3 min; (E, F) 4 min. When the reaction is terminated one minute after it starts, no nucleation process occurs and the surface of Fe_3_O_4_ nanoparticles remains unchanged. Extending the reaction time, isotropic nucleation (coating on the surface of Fe_3_O_4_ nanoparticles) and anisotropic nucleation (mSiO_2_ nanorod growth) of silane occur simultaneously.


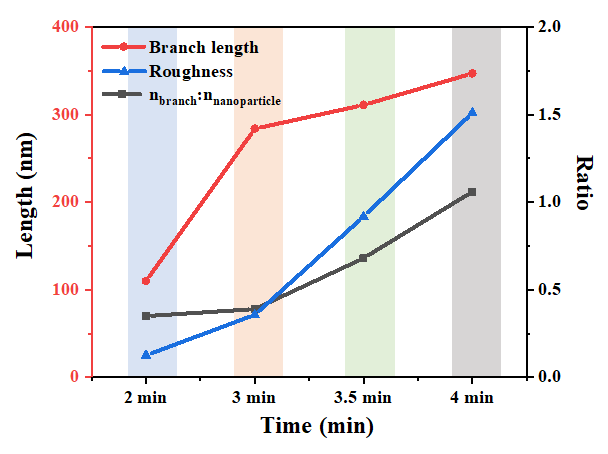
**Figure S17.** Statistical charts depicting the correlations between the time of magnetic field introduction and three parameters: branch length, roughness, and the ratio of branches to nanoparticles.

**
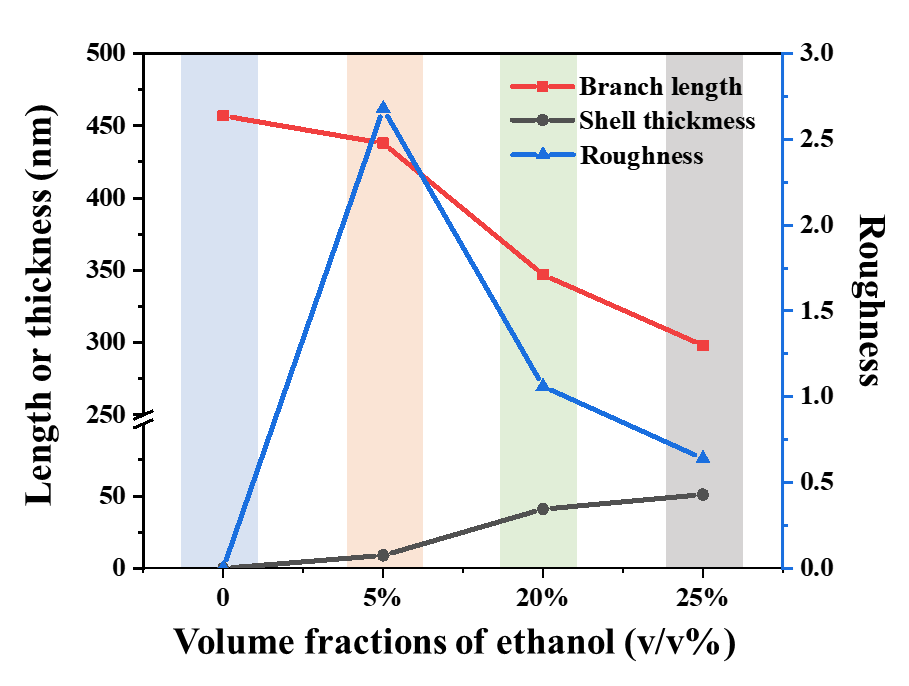
Figure S18.** Statistical charts depicting the correlations between the volume fractions of ethanol (v/v%) and three parameters: branch length, shell thickness and roughness.


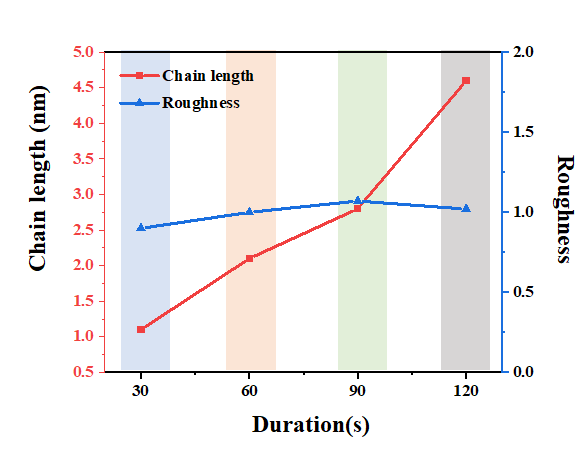
**Figure S19.** Statistical charts depicting the correlations between the duration of magnetic field introduction and two parameters: chain length and roughness.


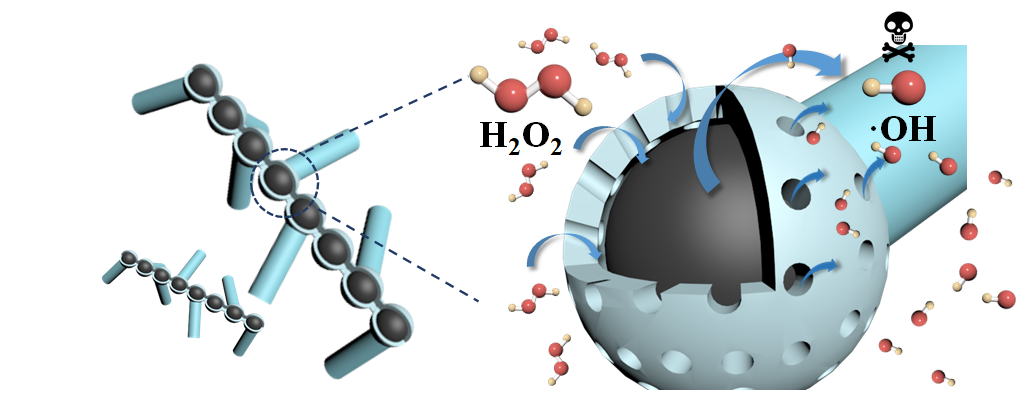


**Figure S20.** Schematic illustration of Fenton reaction based on the core@shell Fe_3_O_4_@mSiO_2_ nanostructure of BNCs. Leveraging the interconnected mesopore channels, Fe_3_O_4_ nanoparticles can react with H_2_O_2_ to generate ROS through the Fenton reaction for antibacterial purposes.

**
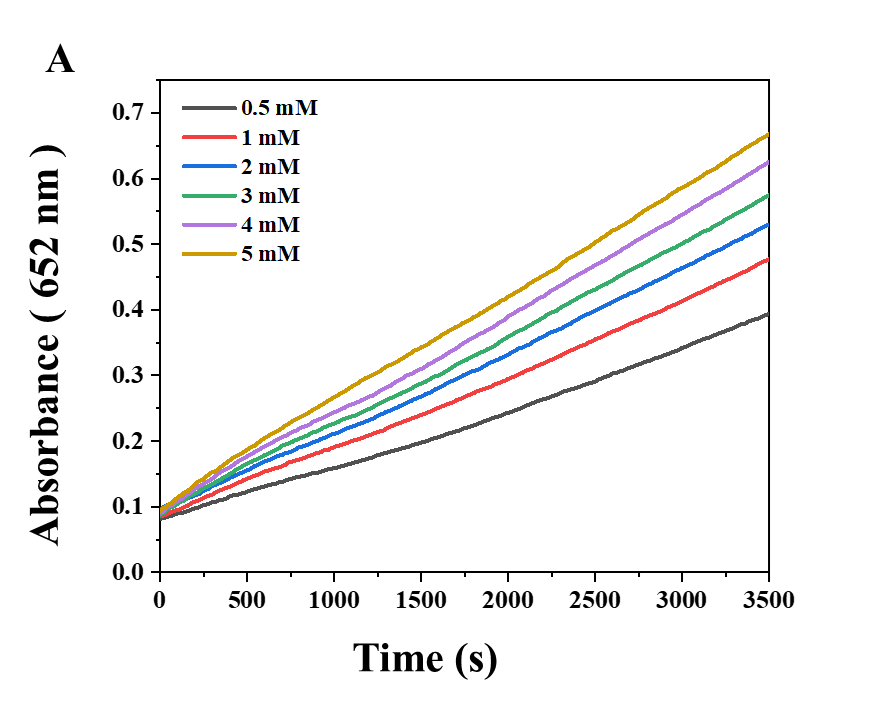
Figure S21.** Time-dependent absorbance changes at 652 nm of oxTMB products under the catalysis of Fe_3_O_4_&mSiO_2_ branched nanochains with different H_2_O_2_ concentrations in the PBS buffer (pH 5.5). The catalytic activity is dependent on the concentration of H_2_O_2._


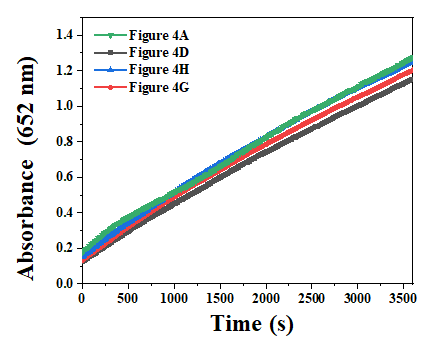
**Figure S22.** Time-dependent absorbance changes at 652 nm of oxTMB products under the catalysis of BNCs of different roughnesses in the PBS buffer (pH 4.0). It can be seen that, despite the varying degrees of exposed Fe_3_O_4_ particles in magnetic chains with different roughness, there is no significant difference in the efficiency of ROS production. This is attributed to the fact that the mesopore channels of mesoporous silica do not significantly affect mass transfer.

**
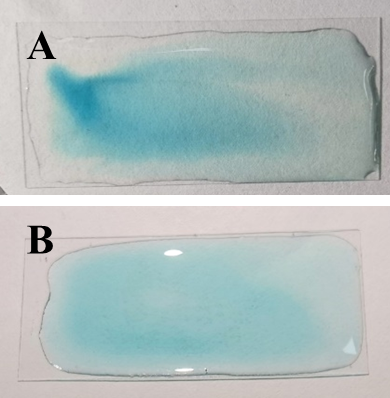
Figure S23.** Photographs of the diffusion condition of oxTMB products without (A) and with (B) the rotating magnetic field (RMF) in the presence of Fe_3_O_4_&mSiO_2_ branched nanochains. The uniform distribution of blue products oxTMB indicates that the rotating magnetic field accelerates the fast and uniform diffusion of the free radicals.

**
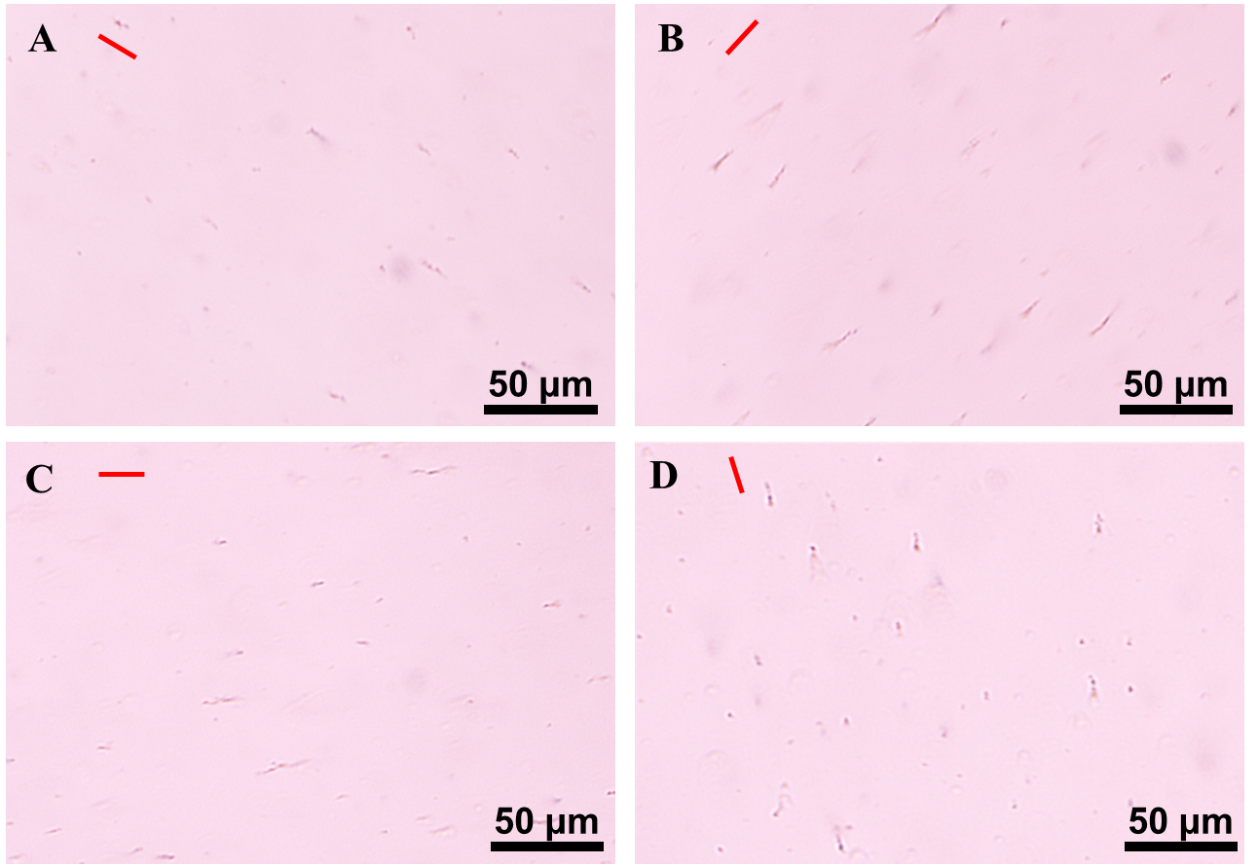
Figure S24.** Photographs of Fe_3_O_4_&mSiO_2_ branched nanochains deflecting under rotating magnetic fields. It can be clearly seen that as the direction of the magnetic field changes, the Fe_3_O_4_&mSiO_2_ branched nanochains rotate with the magnetic field. This demonstrates the availability of the Fe_3_O_4_&mSiO_2_ branched nanochains.

**Figure S25.** OD600 values of the bacteria incubated with different roughness of BNCs. Error bars are taken from three parallel tests per group. Data are expressed as mean standard ± s.d. (n = 3)


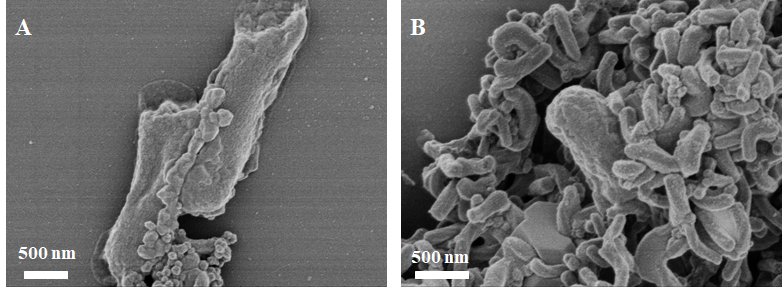


**Figure S26.** SEM images of the bacteria incubated with different roughness of BNCs. ^[2]^ (A) BNCs shown in Figure 4D; (B) BNCs shown in Figure 4G.


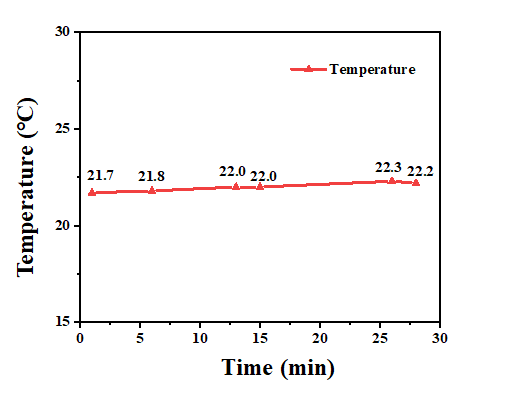
**Figure S27.** Temperature changes in the solution before and after the introduction of RMF at room temperature. The introduction of the RMF did not lead to significant temperature changes.

**
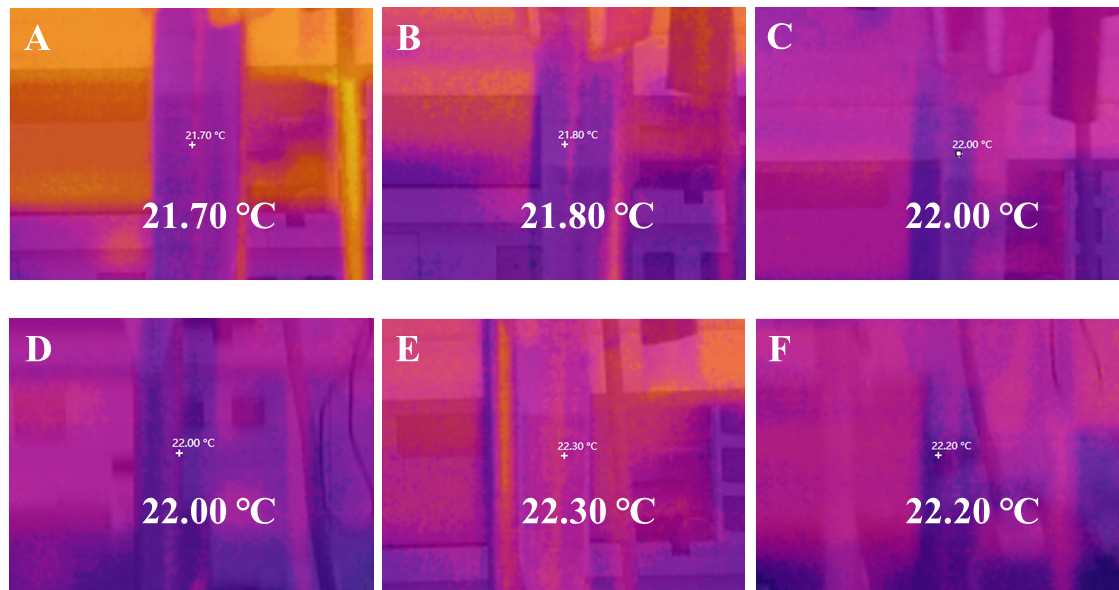
**

**Figure S28.** Infrared thermographic images of bacteria and BNCs co-incubated under RMF conditions at room temperature. The introduction of the RMF did not lead to significant temperature changes.


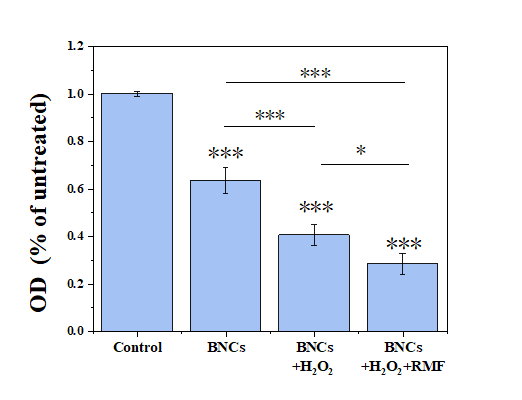
**Figure S29.** OD600 values of bare bacteria and the bacteria incubated with BNCs, BNCs+ H_2_O_2_, and BNCs+ RMF+ H_2_O_2_ groups. Error bars are taken from three parallel tests per group. Data are expressed as mean standard ± s.d. (n = 3)


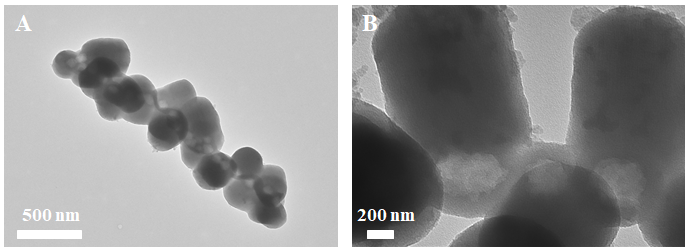


**Figure S30.** TEM images of the pure SiO_2_ rough chains with etched Fe_3_O_4_. After etching Fe_3_O_4_, the BNCs still exhibit a clear rough one-dimensional structure.


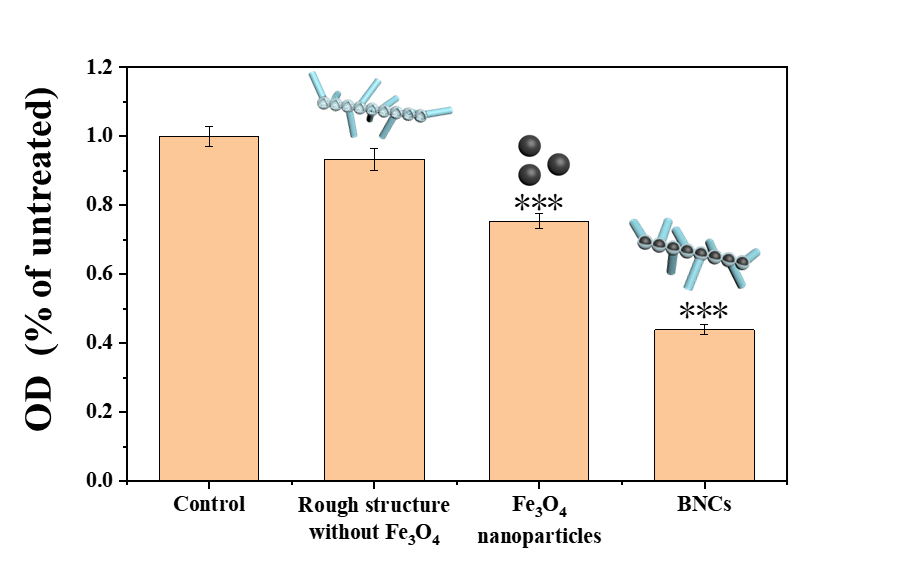
**Figure S31.** OD600 values of bare bacteria and the bacteria incubated with BNCs, dispersed Fe_3_O_4_ nanoparticles and the pure SiO_2_ rough chains with etched Fe_3_O_4_ groups under H_2_O_2_ and RMF conditions. Error bars are taken from three parallel tests per group. Data are expressed as mean standard ± s.d. (n = 3)

**
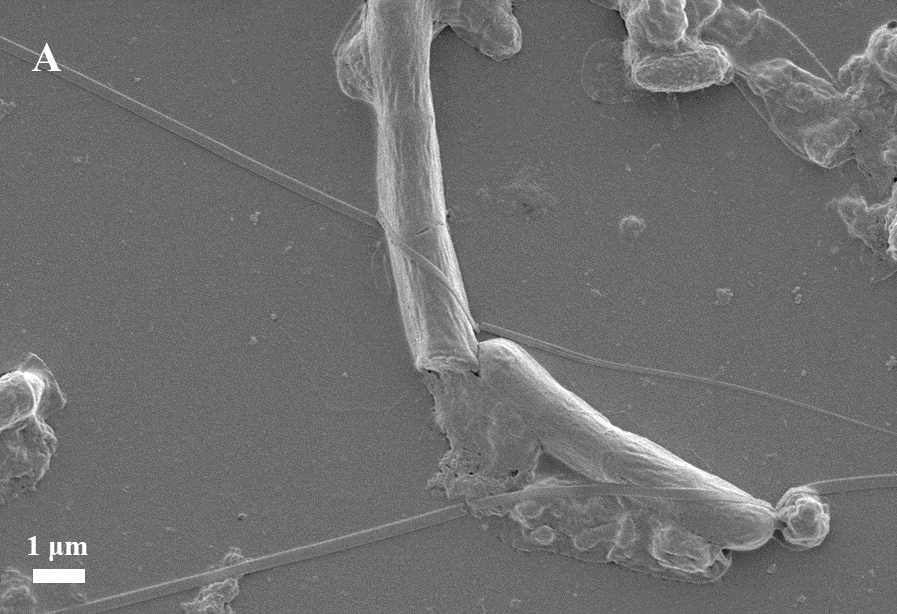
Figure S32.** SEM image of untreated *E. coli* samples. Untreated *E. coli* cells present a typical rod with a smooth surface and possess intact cell walls.

**
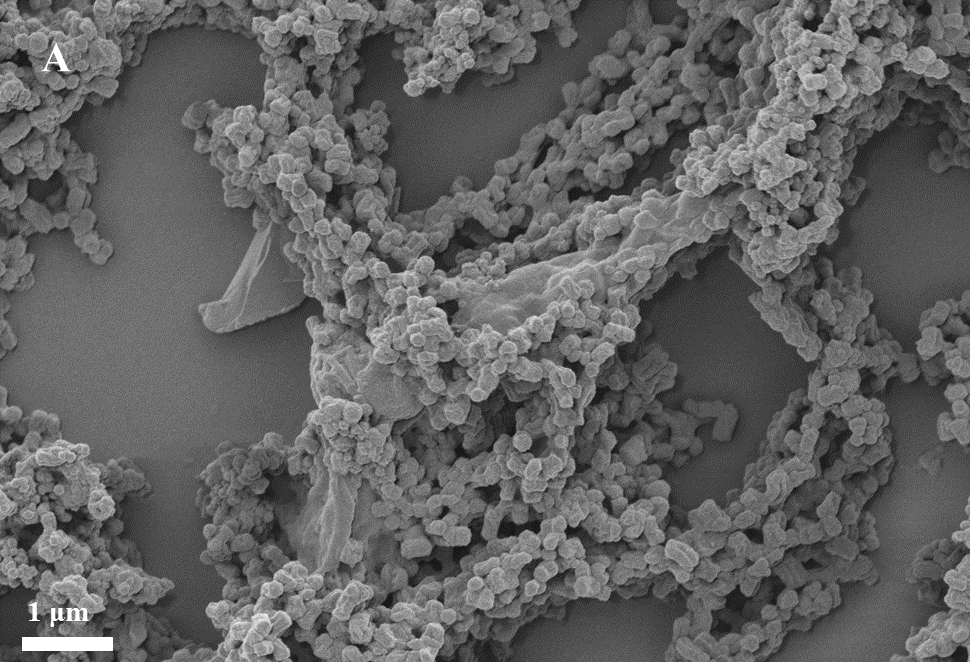
Figure S33.** SEM image of untreated *E. coli* samples exposed to the BNCs + H_2_O_2_ + RMF. This result further demonstrates that a significant number of Fe_3_O_4_&mSiO_2_ branched nanochains adhere to the bacterial surface, resulting in severe damage to the bacteria.

**
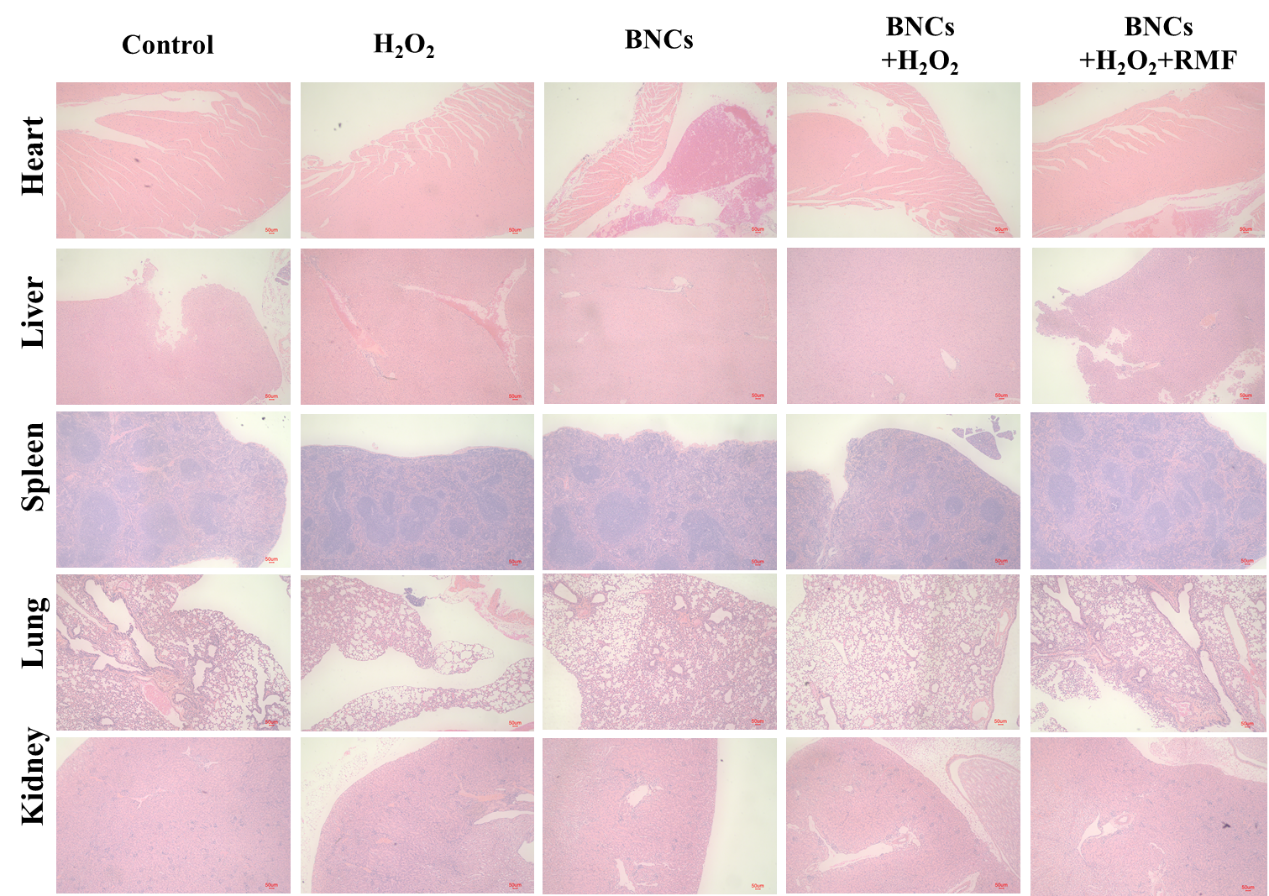
Figure S34.** H&E staining images of visceral tissue slices of the BALB/c mice with different treatments after four days. It is suggested that side effects on major organs are negligible. ^[3]^

**References**

[1] Y. Tang, Y. Liu, W. Li, Y. Xie, Y. Li, J. Wu, S. Wang, Y. Tian, W. Tian, Z. Teng, G. Lu, *RSC Adv.* **2016**, 6, 62550.

[2] F. Cao, L. Zhang, H. Wang, Y. You, Y. Wang, N. Gao, J. Ren, X. Qu, *Angew. Chem. Int. Ed.* **2019**, 58, 16236.

[3] Y. Yang, X. Wu, L. Ma, C. He, S. Cao, Y. Long, J. Huang, R. D. Rodriguez, C. Cheng, C. Zhao, L. Qiu, *Adv. Mater.* **2021**, 33, e2005477.
